# Spatiotemporal variability of nitrogen dioxide (NO_2_) pollution in Manchester (UK) city centre (2017–2018) using a fine spatial scale single-NO_*x*_ diffusion tube network

**DOI:** 10.1007/s10653-021-01149-w

**Published:** 2021-11-05

**Authors:** Daniel Niepsch, Leon J. Clarke, Konstantinos Tzoulas, Gina Cavan

**Affiliations:** Department of Natural Sciences, Faculty of Science and Engineering, Manchester Metropolitan University, Chester Street, Manchester, M1 5GD UK

**Keywords:** Urban air quality, NO_2_, Manchester (UK), Passive diffusion tube sampling

## Abstract

Nitrogen dioxide (NO_2_) is linked to poor air quality and severe human health impacts, including respiratory and cardiovascular diseases and being responsible annually for approximately 23,500 premature deaths in the UK. Automated air quality monitoring stations continuously record pollutants in urban environments but are restricted in number (need for electricity, maintenance and trained operators), only record air quality proximal to their location and cannot document variability of airborne pollutants at finer spatial scales. As an alternative, passive sampling devices such as Palmes-type diffusion tubes can be used to assess the spatial variability of air quality in greater detail, due to their simplicity (e.g. small, light material, no electricity required) and suitability for long-term studies (e.g. deployable in large numbers, useful for screening studies). Accordingly, a one passive diffusion tube sampling approach has been adapted to investigate spatial and temporal variability of NO_2_ concentrations across the City of Manchester (UK). Spatial and temporal detail was obtained by sampling 45 locations over a 12-month period (361 days, to include seasonal variability), resulting in 1080 individual NO_2_ measurements. Elevated NO_2_ concentrations, exceeding the EU/UK limit value of 40 µg m^−3^, were recorded throughout the study period (*N* = 278; 26% of individual measurements), particularly during colder months and across a wide area including residential locations. Of 45 sampling locations, 24% (*N* = 11) showed annual average NO_2_ above the EU/UK limit value, whereas 16% (*N* = 7) showed elevated NO_2_ (> 40 µg m^−3^) for at least 6 months of deployment. Highest NO_2_ was recorded in proximity of highly trafficked major roads, with urban factors such as surrounding building heights also influencing NO_2_ dispersion and distribution. This study demonstrates the importance of high spatial coverage to monitor atmospheric NO_2_ concentrations across urban environments, to aid identification of areas of human health concern, especially in areas that are not covered by automated monitoring stations. This simple, reasonably cheap, quick and easy method, using a single-NO_*x*_ diffusion tube approach, can aid identification of NO_2_ hotspots and provides fine spatial detail of deteriorated air quality. Such an approach can be easily transferred to comparable urban environments to provide an initial screening tool for air quality and air pollution, particularly where local automated air quality monitoring stations are limited. Additionally, such an approach can support air quality assessment studies, e.g. lichen or moss biomonitoring studies.

**Supplementary Information:**

The online version contains supplementary material available at 10.1007/s10653-021-01149-w.

## Introduction

Urban air pollution is a worldwide concern, and urban populations are increasingly exposed to a large number of airborne pollutants that affect human health, such as respiratory and cardiovascular diseases (Schraufnagel et al., 2019). Indeed, poor air quality and air pollution are linked to 40,000 premature deaths in the UK each year, of which 23,500 can be attributed to NO_2_ alone (DEFRA & PHE, 2017; The Royal College of Physicians, 2016).

Nitrogen oxides (NO_*x*_), combining nitric oxide (NO) and nitrogen dioxide (NO_2_), are released into urban environments from combustion processes, such as heating, energy production and road traffic vehicle emission (DEFRA, 2017a). In particular, diesel-vehicle emissions are responsible for large quantities of nitrogen compounds in urban areas, especially when moving slowly (Air Quality Expert Group, 2004; Beckwith et al., 2019). Furthermore, the diesel emission scandal of diesel car manufacturers that incorrectly showed improved performance in pollutant reduction highlighted the potential increased damage to human health (Beckwith et al., 2019; Brand, 2016; Oldenkamp et al., 2016). NO_2_ as a major airborne pollutant is related to adverse health effects (DEFRA, 2017a), of which short- or long-term exposure can have a variety of deleterious health impacts, including respiratory disorders, such as reduced lung function, asthma and bronchitis (Schraufnagel et al., 2019; The Royal College of Physicians, 2016). For instance, Achakulwisut et al. (2019) reported that 19% of childhood asthma within the UK is related to air pollution, especially by NO_2_. Notably, long-term NO_2_ exposure (at or below the current EU/UK regulatory value of 40 µg m^−3^) is responsible for reduced life expectancy by an average of 5 months (DEFRA & PHE, 2017; Regan, 2018; The Royal College of Physicians, 2016).

Due to technical improvements and reduced emissions from road transport and power stations, UK NO_*x*_ emissions have decreased during the last decades (NAEI, 2018a). Comparably, the NO_2_ fraction of NO_*x*_ emissions at roadsides also decreased (Carslaw et al., 2016, 2019; Grange et al., 2017), but exceedances above the UK/EU permissible limit of 40 µg m^−3^ are still frequently observed in urban environments (Beckwith et al., 2019; Carslaw et al., 2011). NO_2_ is primarily emitted at ground level, with subsequent impeded dispersion due to the occurrence of buildings within urban environments being particularly significant for narrow streets and at major road junctions (Cape et al., 2004; Davies et al., 2007). Consequently, urban populations are exposed to NO_2_ as pedestrians or vehicle passengers, most often coinciding with periods of high road traffic, e.g. during commuting times, which result in high short-term exposure, potentially exceeding recommended limits with increased negative health impacts (Beckwith et al., 2019; Molle et al., 2013). Following European Union (EU) legislation, the *Ambient Air Quality Directive* (2008/50/EC), the UK incorporated legally binding limits for outdoor air pollutants, such as NO_2_ annual mean concentration of 40 µg m^−3^ into national law (i.e. the UK Air Quality Standard Regulations; EU, 2008). These binding limits require local authorities to monitor air quality, outline air quality management areas (AQMA) and implement air quality action plans (AQAP). A total of 627 AQMAs for NO_2_ are assigned UK-wide, with most being established in urban areas where frequent EU/UK limit value exceedances (> 40 µg m^−3^) occur (DEFRA, 2017a). For instance, Manchester (UK) was identified as an area of concern due to elevated NO_2_ levels (DEFRA & DfT, 2017; TfGM & GMCA, 2016). Continuous air quality monitoring stations record airborne pollutants, including NO_2_ across UK urban environments; however, these stations are restricted in number (only two located in Manchester city centre) and therefore only record localised air quality. Consequently, currently there is a lack of data documenting the finer spatial scale variability in NO_2_ and air quality within Manchester city centre.

Additional monitoring approaches, such as passive air sampling devices, offer the possibility to achieve finer spatial detail of air pollution, using multi-point sampling methods over larger areas that can support automated air quality measurement programmes (Kot-Wasik et al., 2007; Zabiegała et al., 2010). For instance, Palmes-type diffusion tube samplers coated with triethanolamine (TEA) adsorbent allow determination of NO_2_ concentrations in ambient air (Buzica & Gerboles, 2008). They can replace higher cost equipment (e.g. pumps and power supplies) as they are relatively light, small, simple to use, comprise low-cost material and can be analysed relatively quickly and easily (Cape, 2009; Kot-Wasik et al., 2007; Pienaar et al., 2015). Palmes-type diffusion tubes, originally developed in the 1970s for monitoring workplace exposure (Palmes et al., 1976), have been widely applied for spatial and temporal monitoring of atmospheric NO_2_ concentrations across Europe, the UK and Manchester previously (Cape, 2009; DEFRA, 2017a; TfGM, 2016). While automated continuous monitoring stations provide ‘local’ air quality information, elevated pollutant levels can be expected to occur elsewhere within the complex city structure, thus requiring additional more detailed investigations to outline areas of deteriorated air quality and provide information to effectively reduce and manage air pollution and associated human health impacts. In particular, anthropogenic NO_2_ emissions from vehicular, domestic and commercial emissions (DEFRA, 2017a) are likely to vary across urban environments, according to urban structure and therefore highlight the necessity to assess air quality at a finer spatial resolution.

This study assessed the finer scales of spatial variability of NO_2_ concentrations across Manchester city centre, by deploying Palmes-type diffusion tubes (hereafter diffusion tubes) over a 12-month period (temporal variability), with 2-week duration sampling intervals. A single tube per location sampling approach was used, resulting in 1080 individual NO_2_ concentration measurements at 45 stations (within ~ 200 m of each other), across an area of approximately 10 km^2^. This work was part of a larger lichen biomonitoring research project to undertake a high spatial resolution assessment of air quality across Manchester (UK) city centre and was used to ground-truth lichen nitrogen contents (*N* wt%) (Niepsch, 2019). Lichen tissue *N* wt% reflects nitrogen deposition, and elevated lichen *N* wt% suggests elevated atmospheric nitrogen compounds (i.e. by NO_2;_ Boltersdorf & Werner, 2014)_._ For Manchester, *N* wt% in *X. parietina* ranged between 1.01 and 3.77 wt% (Tab. S1*;* Niepsch, 2019), which is comparable to *N* wt% reported for lichens (including *X. parietina*) in anthropogenic influenced areas, e.g. urban and highly trafficked (Bermejo-Orduna et al., 2014; Boltersdorf & Werner, 2013; Boltersdorf et al., 2014; Gombert et al., 2003).

The NO_2_ dataset has been compared to automated NO_2_ measurements to ascertain validity of the passively derived concentrations. Diffusion tube NO_2_ concentrations also were assessed in terms of spatial and temporal (including seasonal) variability, possible controlling factors (e.g. road traffic volume, proximity to major roads and building height). The NO_2_ dataset was also evaluated in relation to UK/EU limit value (40 µg m^−3^) exceedances, to investigate NO_2_ concentrations that may pose a human health risk in Manchester city centre. For instance, the case of Ella Adoo-Kissi-Debrah, a 9-year-old girl, whose death was attributed to poor air quality and air pollution (including NO_2_; BBC, 2020), highlights the necessity to investigate NO_2_ pollution on a fine spatial scale to tackle deteriorated air quality and air pollution and protect human health. Consequently, using a single-NO_*x*_ diffusion approach, which is accessible and transferable to comparable urban environments, could provide an initial screening tool and support for air quality assessment studies (e.g. biomonitoring) and facilitate air quality improvement and air pollution reduction plans by local authorities.

## Materials and methods

### Study area

The Greater Manchester urban agglomeration in the north-west of England is the second largest UK urban centre. The City of Manchester is the centre of this conurbation, covering an area of ca. 11,500 hectares, with an estimated 566,000 inhabitants (in 2018; Manchester City Council, 2019). Manchester has the highest rate of premature deaths in England for cardiovascular and respiratory diseases and cancer and is also the highest ranked local authority for overall premature deaths (Manchester City Council, 2019). Moreover, Manchester childhood hospital admissions for asthma are ranked first in England and emergency ‘Chronic Obstructive Pulmonary Disease (COPD)’ hospital admissions are ranked fourth in England (> 2× national rate), which illustrate major public health issues that are most likely linked to poor air quality (Academy of Science of South Africa et al., 2019; Regan, 2018).

**Fig. 1:**
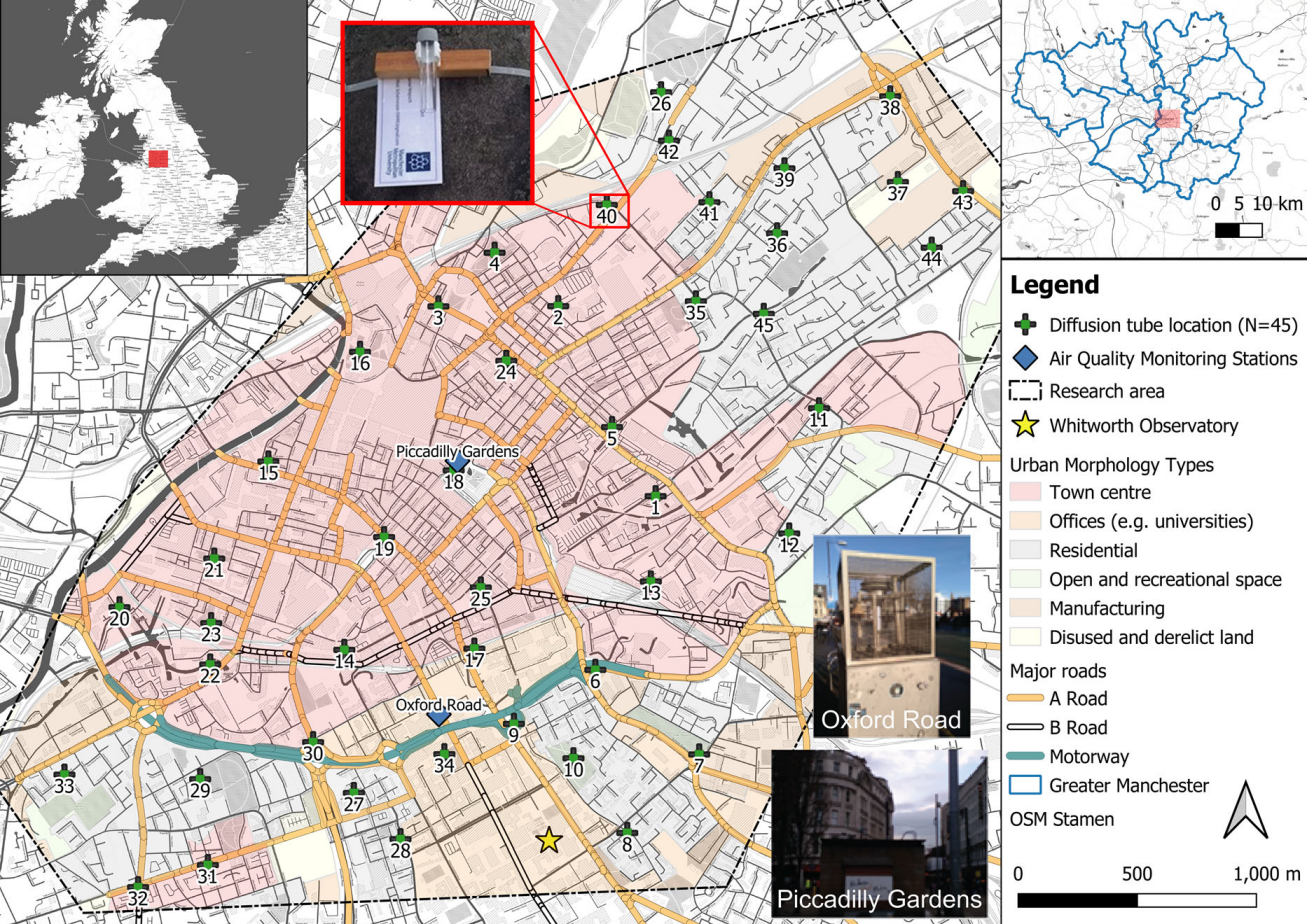
Diffusion tube deployment locations (*N* = 45, with site ID; XY-coordinates in Tab. S1), displayed with automated monitoring stations (Oxford Road and Piccadilly Gardens), meteorological station (yellow star: Whitworth Observatory), urban morphology/land-use type and major road (motorway, A and B road) network in Manchester city centre. Location of research area within Greater Manchester and within the UK is also shown

Manchester city centre falls into the air quality management area (AQMA) outlined by the local authority to ensure improvements in air quality (TfGM, 2016). Hence, two automated monitoring stations are located within the city centre and AQMA and continuously record NO_2_: Piccadilly Gardens (Latitude: 53.481520, Longitude: − 2.237881) and Oxford Road (53.472077, − 2.239001; Fig. 1). Manchester Piccadilly Gardens, an urban centre location (located 200 m away from nearest major road; DEFRA, 2018), broadly represents city-wide background conditions (i.e. in urban residential areas; Loader, 2006). In contrast, Manchester Oxford Road is classified as urban traffic site and is located within 5 m of the kerbside one of Manchester’s busiest roads (Oxford Road; Martin et al., 2011; Regan, 2018). Notably, Oxford Road monitoring station continuously records elevated NO_2_ (2010–2019), often reaching 70–80 µg m^−3^ during most winters and peak values of 97 µg m^−3^ and 100 µg m^−3^ in December 2010 and November 2016 (Fig. S2b). A comparable NO_2_ trend was recorded at Piccadilly Gardens, reaching 40–50 µg m^−3^ during winter months and between 30 and 40 µg m^−3^ during warmer summer months (Fig. S2b). While ‘Clean Air Greater Manchester’ does provide a passive diffusion tube network across Greater Manchester to supplement automatic monitoring stations (GMCA & TfGM, 2019), only 11 diffusion tube locations (out of 400 for Greater Manchester) are located within this study’s research area (Manchester city centre; ~ 10 km^2^), indicating the limitations of high spatial resolution assessment of air quality as provided by this study (i.e. at 45 sites).

The research area is a SW–NE transect across central Manchester (Fig. 1) and includes different land-use types, e.g. town centre and retail, residential and industrial areas, as well as open green spaces. The city centre is characterised by higher buildings, increased traffic numbers, including public transport (buses, trams and trains) and slow traffic movement, particularly during peak hours (AM and PM peak; Highway Forecasting an Analytical Services, 2015).

### Diffusion tube procedure

Selection of diffusion tube deployment sites was based on assessment of lichen nitrogen contents (Tab. S1; *N* = 94 for *X. parietina*; *N* wt% obtained by an LECO TruSpec CN elemental analyser; Niepsch, 2019), which indicated spatial variability of airborne nitrogen compound concentrations across Manchester, i.e. by NO_2_ (Boltersdorf & Werner, 2014). Deployment sites were systematically selected across the range of lichen *N* wt% (Tab. S1) and to maintain approximately 200 m between each sampling location to obtain a high spatial resolution NO_2_ sampling approach.

A modified practical guidance, published by the UK Department for Environment, Food & Rural Affairs (DEFRA), was used to prepare, deploy, clean and re-charge diffusion tubes (DEFRA, 2008). To prepare diffusion tubes, stainless-steel meshes were soaked in a triethanolamine (TEA)/acetone (v:v; 1:1) solution for 1 h and then dried on a paper towel for 15 min. For each tube, two impregnated and dried meshes were placed in the grey cap and the acrylic diffusion tubes were capped at the other end with a beige-coloured cap. Assembled diffusion tubes were refrigerated in sealed plastic bags until deployment and between recovery and IC analysis (Sect. 2.3). Laboratory blanks and travel blanks, consisting of prepared and assembled diffusion tubes, were included and handled in the exact same way as deployed tubes (DEFRA, 2008). Laboratory blanks (*N* = 3) for each diffusion tube deployment batch were kept refrigerated (unexposed) in zip-lock bags and analysed with exposed tubes to control for potential contamination. Laboratory blank nitrite (NO_2_^−^) concentrations were used for ‘blank subtraction’, separately for each deployment batch, before data analysis of exposed diffusion tubes. In contrast, travel blanks (*N* = 1 for each batch) were carried during deployment (prepared and kept within zip-lock bags), but not exposed, to identify any possible contamination of tubes while in transit or in storage (DEFRA, 2008). Atmospheric NO_2_ concentrations were calculated according the DEFRA protocol (DEFRA, 2008) as summarised in Tab. S5.

Diffusion tubes (Gradko International, UK; one per site) were deployed on 45 urban trees (Fig. 1), at heights of 2.0–2.5 m above ground to avoid vandalism. Tubes faced towards the closest road and were fixed on site with re-usable plastic straps, mounting clips and a spacer block (no protective housing was used; Fig. 1) away from vertical surfaces to ensure free circulation of air (DEFRA, 2008). Deployments started on 3 July 2017 for a ca. 12-month period until 28 June 2018 (361 days), with tubes being changed every 2 weeks (actual deployment dates in Tab. S2). It is recommended to expose diffusion tubes in replicates (ideally triplicates) to obtain more robust results (Cape, 2009; DEFRA, 2008). However, due to resource limitations (e.g. only one fieldworker/analyst), the high spatial resolution approach adopted (*N* = 45 sites), 2-weekly tube changes (fieldwork workload for one person) and large total number of tubes for IC analysis (including prior extraction procedure), only one tube was deployed per site.

Deployed diffusion tubes were extracted at the end of a 2-week deployment period (i.e. a batch of samples), using 3 mL of ultrapure water (18.2 MΩ) for 30 min, then filtered through 0.2-µm nylon filters (Fisherbrand™ non-sterile nylon syringe filter, Fisher Scientific, UK) and subsequently analysed on a Thermo Scientific—ICS5000 ion chromatography (IC) system (Thermo Fisher, UK, Sect. 2.3). Analysed diffusion tubes and stainless-steel meshes then were separated, and cleaning comprised an ultrasonic bath for 15 min with cleaning agent ‘Decon 90’ (Camlab, UK). Prior to ultrasonication, tube components were soaked in cleaning agent and deep cleaned with cotton-tipped brushes. Steel meshes were additionally washed with 1 mol L^−1^ hydrochloric acid (Fisher Chemical™, Fisher Scientific, UK). Steel meshes and tube components were triple rinsed with ultrapure water, and steel meshes were oven-dried at 100 °C. Dried components were stored in sealed plastic bags (steel meshes in tin foil capped beaker) until reassembling prior to future redeployment.

**Fig. 2:**
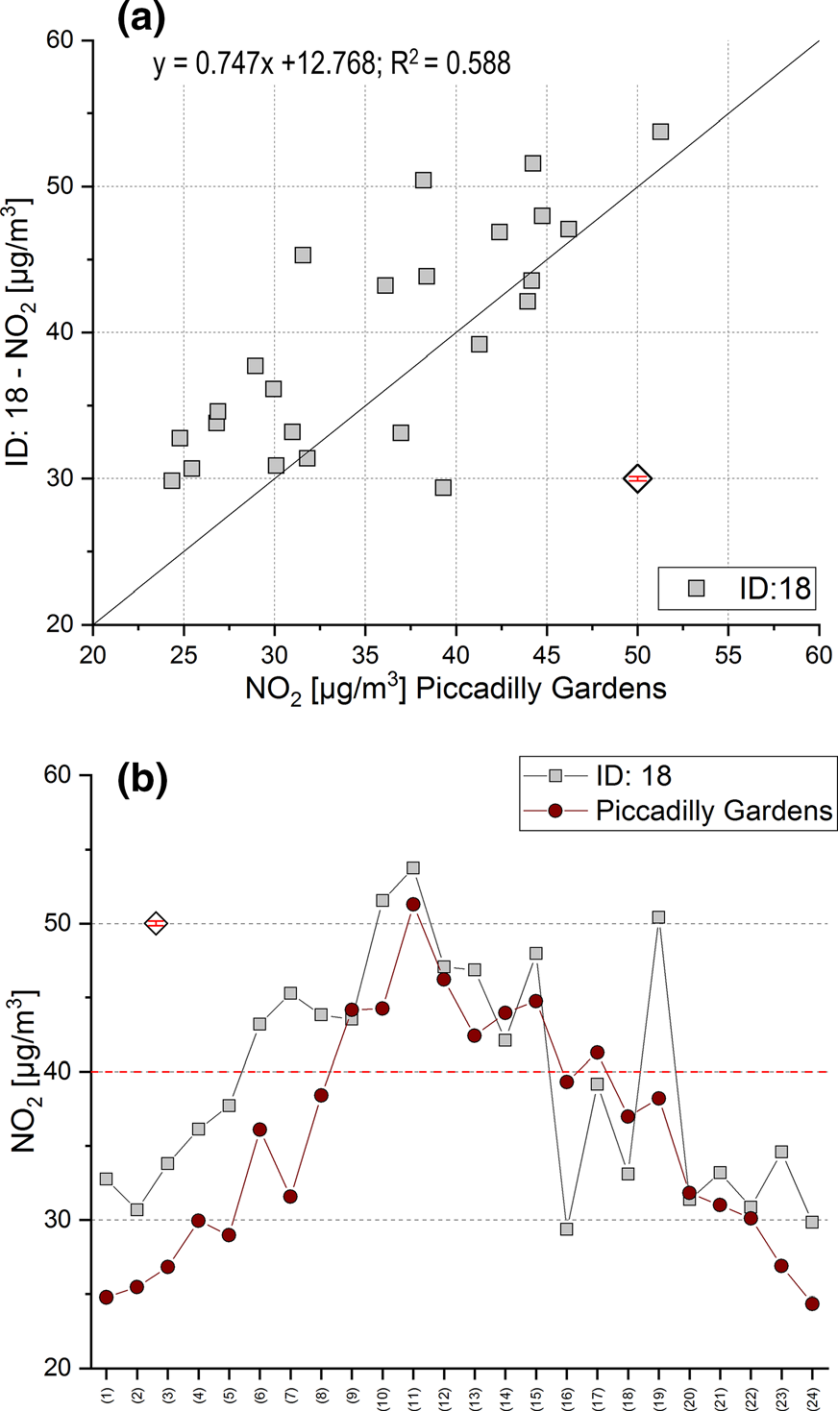
Comparison of bi-weekly (14 days) diffusion tube (ID: 18) and automated NO_2_ concentration measurements at Piccadilly Gardens [calculated as mean NO_2_ for the same bi-weekly period; (**a**) and (**b**)]; **a** linear regression equation and *R*^2^ and **b** IC nitrite measurement errors (CRM—Simple Nutrients: NO_2_^−^) plotted on a dummy value (white diamond)

To validate diffusion tube NO_2_ data, a ‘co-location’ study with the Piccadilly Gardens automated air quality monitoring station was undertaken (Fig. 1). Direct deployment of diffusion tubes adjacent to the automated samplers was not possible, due to necessity of requiring permissions to access the monitoring station and measurement inlet on top of the monitoring station (at a height of 4 m). Hence, the closest diffusion tube deployment location (i.e. street tree) was used for comparison, i.e. ID: 18 (Fig. 2; Table S6). This location was also sampled for lichen material (*X. parietina*), and nitrogen contents (2.95 N wt%; Tab. S1) suggested elevated ambient nitrogen concentrations. The diffusion tube site used for comparison was located ~ 10 m away from the automated monitoring station. A comparison with other passive NO_2_ sampling devices (e.g. badge-type samplers) was out of scope for this study, due to the use of diffusion tubes to assess spatial and temporal variability of NO_2_ and ground-truth lichen *N* wt% data (Niepsch, 2019).

### Analysis of extracted diffusion tubes by ion chromatography (IC)

Ion chromatography (Thermo Scientific ICS-5000) equipment characteristics for anions (nitrite—NO_2_^−^) were: AG18 (2 mm × 5 mm) guard column, AS18 (2 mm × 250 mm) separation column; EGC III KOH cartridge (electronically generated elution; potassium hydroxide—KOH), starting at 18 mM KOH (slope: 1.96 mM/min) for 16 min. The signal was measured using suppressed conductivity. IC calibration standards containing 0, 0.05, 0.1, 0.5, 1.0 and 2.0 µg mL^−1^ nitrite (NO_2_^−^) were made up from ‘Dionex™ Seven Anion Standard II’ (Thermo Fisher, UK), fresh for each analytical batch. Calibration linearity (*R*^2^ > 0.98) was checked and confirmed before data processing for each analytical batch. Certified reference material (CRM) ‘Simple Nutrients—Whole Volume (QC3198; Sigma-Aldrich, UK)’ was used to assess IC accuracy and precision throughout the study (*N* = 230) and to check for any batch-to-batch variability in data quality. The CRM contained NO_2_^−^ as *N* (certified value: 2.95 ± 0.0536 mg L^−1^), which had to be converted to NO_2_^−^ using a factor of 3.28443 (based on IUPAC 2019 atomic weights for nitrogen and oxygen) to allow comparison with IC NO_2_^−^ measurements (CRM-NO_2_^−^ = 9.69 mg L^−1^; CRM was diluted by a factor of 5). Nitrate as N converted to NO_3_^−^ (using a factor of 4.42664) concentrations were used to assess potential oxidation of NO_2_ to NO_3_ during the use of the CRM and potential influences on NO_2_^−^ accuracy. CRM-NO_2_^−^ varied between 99 and 120% within analytical batches (Fig. S4) with higher NO_2_^−^ concentrations during four analytical runs (e.g. 7, 11, 19 and 22; Fig. S4b). Overall, CRM accuracy was 104% (10.07 ± 0.58 mg L^−1^) for NO_2_^−^ and 105% (48.84 ± 6.61 mg L^−1^) for NO_3_^−^, indicating the usability and stability of the CRM during the diffusion tube deployment period. Overall precision (coefficient of variation—%CV) was found at 5.7%, and repeatability of measurements further illustrates the suitability of the analytical method to precisely extract and analyse NO_2_^−^ from diffusion tubes.

IC lower limits of detection (LLD) for NO_2_^−^ were determined using ultrapure water blanks, analysed throughout each analytical run and calculated as three times the standard deviation, resulting in highest LLD for NO_2_^−^ of 0.078 mg L^−1^ for all analytical runs. Analysed NO_2_^−^ concentrations were above the LLD for each batch of tubes.

### Meteorological and auxiliary datasets

Meteorological data (temperature, precipitation, sunshine hours, wind speed and direction) were obtained from the Whitworth Meteorological Observatory (Fig. 1; ‘Whitworth Meteorological Observatory—Data Archive’, 2018; longitude: N53.467374, latitude: W2.232006, altitude: 43 m), for every 2-week deployment period (Tab. S3). Due to deployment of diffusion tubes on urban trees and the potential variation of micro-climatic conditions at sampling locations, data obtained from the Whitworth Observatory were only used to investigate potential meteorological impacts on ‘overall’ diffusion tube performance (Cape, 2009). Micro-climatic conditions at diffusion tube locations that are influenced by the tree itself (e.g. decrease in temperature, Janhäll, 2015; Lee & Park, 2008) and the urban surrounding (e.g. impact on wind velocity and patterns by building density and building heights, Kubota et al., 2008; Oke, 1988) were beyond the scope of this study and were not used to ascertain ‘local’ influences on NO_2_.

Partitioning of NO_2_ measurements to facilitate investigation of ‘seasonal’ variability was completed using six subsequent diffusion tube measurements for each location, i.e. mean of six bi-weekly NO_2_ concentrations (representing three months). Initial diffusion tube deployment began in July 2017 and ended in June 2018 (361 days). Therefore, a subdivision into four strict meteorological seasons was not possible and a shift of seasons by 1 month had to be applied. Subdivision was based on meteorological parameters, in particular temperature (°C) and precipitation (mm; data for bi-weekly deployments at the Whitworth Observatory in Tab. S3, Fig. S1). Temperatures (*T*_min_ and *T*_max_ in °C) for defined ‘seasons’ were comparable to historical data from Manchester (Manchester Ringway, lat.: 53.356, long.: − 2.279, in use 1946–2004), e.g. with July/August being the warmest months (max. temp. °C) and driest (average precipitation, mm; Met Office, 2021). Here, ‘spring’ (April–June) had the highest temperatures and lowest total precipitation (mm, Fig. S1), which is most likely related to the shift in season and the recorded drier months in 2017 and 2018 (Met Office, 2018).

Auxiliary datasets, available via public domain sources (i.e. Digimap—Ordnance Survey and Department for Transport—DfT), were used to investigate potential urban influences on NO_2_ concentrations, including distance to major roads (e.g. motorways and A-roads), traffic counts (‘annual average daily traffic flow’) and surrounding building heights (OS—building heights, alpha release; Digimap - Ordnance Survey, 2017).

Justification of data classifications and data groupings is included in Tab. S4 and Fig. S3. Data classification and grouping was informed by studies focussing on decline of NO_2_ with increased distance from major roads (Bermejo-Orduna et al., 2014; Gilbert et al., 2003; Laffray et al., 2010). Road class groupings followed the ‘primary route network’ classifications used within the UK, including motorway, A roads (major arterial roads), B roads (distributor roads, lower traffic density than A roads) and unclassified (local roads for local traffic; UK Department of Transport, 2012). Distances were measured within GIS from the sampling location to the closest major road (i.e. motorway and A-road).

Traffic count data, as ‘annual average daily traffic flow’ (DfT, 2017), were used to investigate overall traffic influences. AADF data are available as point data for A roads and motorways, produced for each junction-to-junction link on a major road (estimated or counted; DfT, 2017). Due to dynamic traffic movements on roads, i.e. increases and decreases from the estimated or counted point, a 500-m buffer around the sampling site (using GIS) was used to include the road segment as a whole. Moreover, 500-m buffers were used to include potential traffic influences for locations where no data were available (e.g. highly trafficked minor/unclassified roads). If more than one traffic count point was within the buffer, traffic data were averaged separately.

Building heights were analysed using ‘relative height from ground level to highest part of the roof’ (relHmax; Digimap - Ordnance Survey, 2017). A 50-m buffer around the diffusion tube location was used, and the average building height of ‘relHmax’ was calculated within the pre-set buffer, to reflect the closer surroundings of the sampling location. To test whether more restricted air flow around higher buildings is associated with poorer air quality (e.g. recirculation, ventilation and airflow), Manchester’s building heights were categorised into three different groups: open (< 10 m, e.g. residential buildings), medium (10–20 m, e.g. mixed-use buildings) and high (> 20 m, e.g. high-rise buildings; Dobre et al., 2005; Lo & Ngan, 2015; Longley et al., 2004). The UK *Building Act 1984* and the *Building Regulations* 2010 and 2018 were used as guidance for height groups. However, ‘urban street canyon’ effects, i.e. recirculation and wind flow in varying building and street geometry, were beyond the scope of this study due to the particular diffusion tube deployments on urban trees and inherent impacts, and the additional workload required to record micro-climatic conditions and surrounding detail at each tube deployment location.

### Statistical and geospatial data analysis

Data visualisation and statistical testing were performed using Graph Pad Prism 7 and Origin 2019 (GraphPad Software Inc., 2018; Origin Lab 2018). Measured diffusion tube NO_2_ concentrations were normally distributed using Shapiro–Wilk test (Razali & Wah, 2011), favouring parametric statistical analysis. For instance, Pearson’s r (correlation statistics) was used to assess potential relationships between recorded NO_2_ concentrations and urban influencing factors (e.g. major road distance, traffic count data and building heights). NO_2_ concentrations and grouped urban influencing factor data (Fig. S3 and Tab. S4) were compared using ANOVA test statistics. Geographic Information Software (GIS) QGIS 3.10 (QGIS Development Team, 2019) was used for mapping of NO_2_ concentrations.

## Results and discussion

### Comparison of diffusion tube NO_2_ measurements with automated continuous air quality monitoring station records and limitations of diffusion tube measurements

Comparable trends of recorded NO_2_ concentrations were observed by Piccadilly Gardens automated monitoring station and the closest diffusion tube location (ID: 18). Figure 2 shows the comparison of 2-weekly NO_2_ concentration averages (automated and diffusion tube location), for the ca. 12-month duration measurement period. The systematic error (bias) calculated as difference between diffusion tube NO_2_ concentration and ‘true’ automated air quality monitoring station NO_2_ for the deployment period ranged between − 9.95 and 13.69 µg m^−3^. Overall, passively derived NO_2_ concentrations were largely positively biased, when compared to the reference value (Fig. 2a). Nonetheless, a comparable trend between automated and diffusion-tube-derived NO_2_ concentrations was recorded (Fig. 2b). Diffusion tubes are categorised as ‘indicative’ measurements and uncertainty has been quoted as ± 25%, compared to ± 15% for the reference method (DEFRA, 2008; EC, 2008). Bush et al. (2001) reported differences of ± 24–38% for individual diffusion tube measurements, co-located with chemiluminescence analysers (*N* = 17), which is comparable to results for the single-tube approach used in this study, with a percentage difference between single diffusion tube and automated NO_2_ measurements ranging between − 29 and 36% (Bush et al., 2001; Hafkenscheid et al., 2009). However, compared to the reference analyser, 18 out of 24 (75%) bi-weekly diffusion tube-derived NO_2_ measurements were within set limits of ± 25%.

Passive diffusion tubes are well known to have the potential for greater uncertainty than the reference method (i.e. chemiluminescence analysers) and potential biases (over- and/or underestimation of NO_2_ concentrations) may be introduced during the preparation, during exposure (e.g. by environmental factors: wind, temperature and humidity), post-exposure NO_2_^−^ quantification, exposure-average NO_2_ calculation and comparison of diffusion tube measurements with co-location against the reference method as ‘true’ NO_2_ concentration (DEFRA, 2008; EU, 2008; Heal et al., 2019). Because of the one-tube approach applied in this study, potential impacts were carefully considered.

Bias from preparation and extraction effects were most likely to affect diffusion tube performance; for example, the lowest analysed NO_2_ concentration (2 µg m^−3^) could be related to insufficient coating of the meshes with TEA/acetone mixture or insufficient NO_2_^−^ extraction (Cape, 2009; Heal et al., 2019). Potential exposure biases (negative and positive) in diffusion tube determined NO_2_ concentrations in relation to environmental parameters were considered minor, because influencing meteorological conditions, e.g. temperature and wind speed as specified in Cape (2009), were within scope to not impact on tube performance (data from Whitworth Observatory, Fig. S1). Relative humidity less than ~ 75% is reportedly impacting on the NO_2_/TEA conversion to NO_2_^−^ (Heal et al., 2019), which may have resulted in negative bias, but no humidity data at diffusion tube locations were available for comparison. Interferences by co-pollutants (e.g. nitrous acid—HONO, peroxyacetyl nitrate—PAN) and UV light blocking could not be fully excluded; the latter due to fixation of diffusion tubes on urban trees and consequently potential NO_2_ photolysis impacts by tree foliage (Cape, 2009; Fantozzi et al., 2015; Heal et al., 2019). Nevertheless, influences from HONO and PAN under UK conditions are likely to be small (Cape, 2009; Heal et al., 2019).

Overall, comparison of NO_2_ concentrations by passive and active (i.e. continuous) measurements showed comparable NO_2_ concentrations, albeit diffusion tubes showing a general overestimation of NO_2_ (Fig. 2). Such a positive bias is most likely related to impacts from the deployment site (i.e. tree). For instance, vegetation is closely linked to the temperature–humidity system (Janhäll, 2015), factors that can also positively influence diffusion tube measurements (Heal et al., 2019). Further, Salmond et al. (2013) reported a net accumulation of NO and NO_2_ below tree canopies and positive bias on diffusion tube NO_2_ from within-tube chemistry (i.e. additional NO_2_ from NO and O_3_; Heal et al., 2019) could explain the observed differences. In this study, diffusion tubes were deployed between 2 and 2.5 m height on the tree trunk (below the tree canopy), whereas the inlet of the continuous measurement station is at a height of 4 m (DEFRA, 2018), further suggesting potential impacts on recorded NO_2_ by tree foliage. In comparison, negative bias (for bi-weekly comparison) of diffusion tube NO_2_ was recorded during colder seasons, suggesting temperature-related influences (Heal et al., 2019), additionally to effects by urban vegetation (Janhäll, 2015; Salmond et al., 2013). Interception and leaf-uptake of NO_2_ by urban trees and canopy effects (from air flow, and horizontal and vertical dispersion) were beyond this study’s scope, but were reported to vary according to tree species (Fantozzi et al., 2015; Salmond et al., 2013). Moreover, micro-climatic conditions at each diffusion tube deployment location could vary during deployment, due to the amount of vegetation (and other urban factors, e.g. building density) in the surrounding area and thus could not be fully accounted for in this study.

Local bias adjustment factors can be used to calculate accuracy and precision for co-location studies; however, these should be applied to annual averages and are not valid for individual results (i.e. the presented bi-weekly periods), due to varying diffusion tube performance depending on meteorological (and other) factors (DEFRA, 2008). For Manchester, a bias adjustment factor of 0.88 for 2017 has been reported (using the DEFRA National Bias Adjustment Factors Spreadsheet, July 2018, version 06/18; GMCA, 2018), which was not used due to the differences in used tube preparation method, i.e. TEA/acetone (50/50) in this study and 20% TEA in water by the Greater Manchester Combined Authority (GMCA, 2018). Because of the single-tube approach applied, accuracy and precision of replicate diffusion tube measurements could not be used to ascertain a bias adjustment factor for the deployment period and for each individual deployment location.

Notwithstanding the above-described limitations, results presented for this ‘co-location’ study indicate the viability of passively derived NO_2_ concentrations. Albeit using one diffusion tube only that was deployed on urban vegetation, the used approach could provide a quick, initial screening tool to identify areas of elevated NO_2_ (i.e. > 40 µg m^−3^) and to achieve high spatial resolution and investigate NO_2_ in more detail. Consequently, such an approach can be extended using a more robust three-tube monitoring approach (i.e. accounting for between tube variability), to further investigate potential pollution hotspots. Integrating micro-climatic conditions and urban vegetation effects (Salmond et al., 2013) could provide further information on recorded passive diffusion tube NO_2_ concentrations. Additional pollutants, e.g. NO and O_3_ measurements, that are linked to atmospheric conversion to NO_2_ (Clapp & Jenkin, 2001; Vardoulakis et al., 2011) could further improve the understanding of diffusion tube performance during a measurement campaign.

### Spatial variability of NO_2_ concentrations and potential urban layout influences on recorded NO_2_

**Fig. 3:**
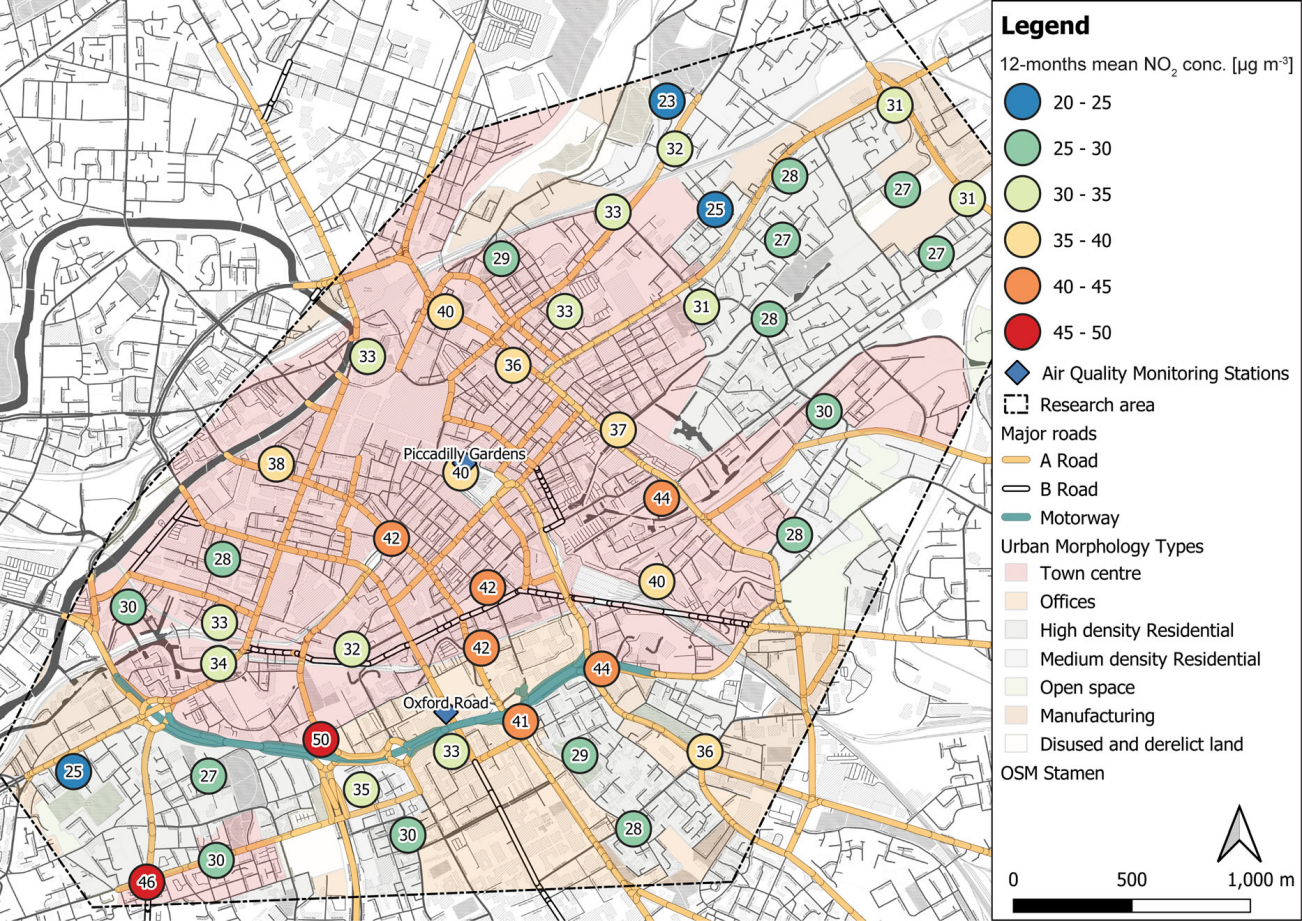
Twelve-month mean NO_2_ concentrations [µg m^−3^] at the 45 diffusion tube locations, deployed across the Manchester city centre (UK), displayed with Urban Morphology Types (UTMs) and major road classes; numbers in circles represent annual (12 months) average NO_2_ concentration at deployment site (viz. at deployment tree)

**Fig. 4:**
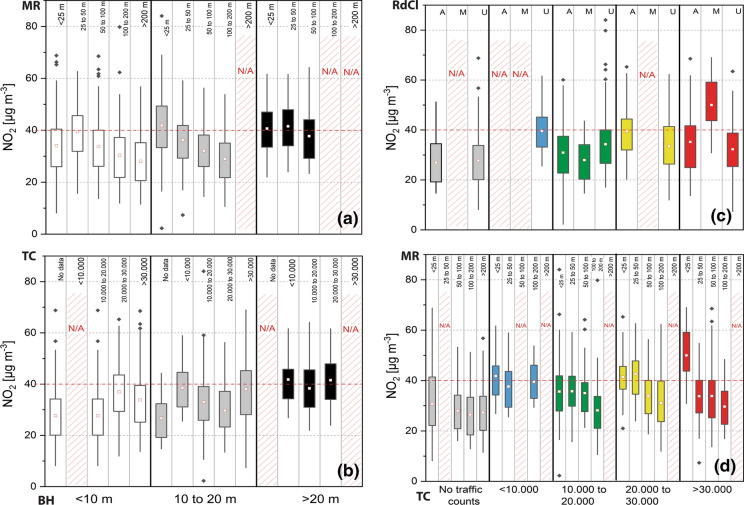
Box plots (IQR—25–75th percentile, whiskers as 1.5*IQR and outliers as black diamonds) of individual NO_2_ concentration measurements analysed by grouped data: **a** building height (BH) and major road distance (MR); **b** building heights (BH) and traffic counts (TC); **c** traffic counts (TC) and road class (RdCl); and **d** traffic count (TC) and major road distance (MR). **a** and **b** Colour coded by building heights, **c** and **d** colour coded by traffic counts; N/A—no data in group; dashed line represents the EU/UK limit value (40 µg m^−3^)

NO_2_ concentrations recorded at sampling sites varied during the 12-month deployment period (Tab. S6; Figs. S5–S12), with individual NO_2_ concentrations ranging from 2.26 to 84.05 µg m^−3^ (Tab. S6; individual NO_2_ for each site, Figs. S5–S12). Higher (mean) NO_2_ occurred alongside the major road network (motorway (M), A-roads (A)) and within the city centre (Fig. 3). NO_2_ hotspots and elevated NO_2_ levels (≥ 30 µg m^−3^) also occurred outside the city centre area, e.g. in less densely built-up and residential areas in the north-east and south-west of the research area (Fig. 3). NO_2_ concentrations declined significantly (*r* =  − 0.61; *p* < 0.01) with distance to major roads, whereas traffic count data (all vehicles and subdivided by vehicle type) did not show such a relationship. Figure 4 illustrates statistically significant differences between recorded NO_2_ concentrations and grouped data for road distances (*F* = 3.69; *p* < 0.05), traffic counts (*F* = 4.17; *p* < 0.01) and building heights (*F* = 3.22; *p* = 0.05); conversely, no significant difference was observed for road class groups (*F* = 0.72; *p* = 0.49). For instance, Fig. 4a, b illustrates potential street canyon effects on recorded NO_2_ concentrations, whereas Fig. 4c, d shows generally higher NO_2_ at roadside locations (e.g. *M*—motorway) across Manchester city centre.

It is a well-known fact that NO_2_ rapidly declines with distance to source (Fig. 4d), e.g. within 200 m from major roads (Bermejo-Orduna et al., 2014; Gilbert et al., 2003; Laffray et al., 2010). However, traffic is named the primary source of NO_2_ in urban centres and at roadside locations across the UK (including Manchester) and other European countries (Bower et al., 1991; Caballero et al., 2012; Casquero-Vera et al., 2019; Hewitt, 1991; Regan, 2018; Vardoulakis et al., 2011). For Manchester, elevated NO_2_ concentrations are mainly associated with arterial roads leading into the city centre and are primarily related to diesel vehicular emissions (Regan, 2018; TfGM, 2016). One site, in particular (ID: 30), showed consistently high NO_2_ concentrations (mean NO_2_: 50 µg m^−3^_;_ 59,000 vehicles daily; DfT, 2017), validating well the understanding that NO_2_ is highest at roadside locations (i.e. within 25 m, Fig. 4d) that can pose a significant threat to human health (Grange et al., 2017).

Elevated NO_2_ concentrations at other roadside locations and road junctions (e.g. 45–50 µg m^−3^, Figs. 3 and 4d < 25 m) are potentially related to particular traffic regimes at diffusion tube deployment sites, due to accelerating, queuing or cruising traffic (Beckwith et al., 2019). Amongst NO, some types of VOCs (e.g., benzene and toluene) are primarily traffic induced and act as precursors for photochemical reactions (e.g. O_3_ and NO_2_; Gentner et al., 2013; Keuken et al., 2012; Kurtenbach et al., 2012; Masiol et al., 2017). For instance, toluene has been a predominant constituent at highly trafficked roads (Parra et al., 2009), which together with nitrogen oxides has a great influence on atmospheric chemistry and air quality (Crutzen, 1995; National Research Council, 1991; Xue et al., 2013). However, VOC concentrations in northern European cities are generally lower compared to those in the south (Parra et al., 2009). Traffic speed within Manchester’s city centre was reported to be below 10 mph (16 km h^−1^; Highway Forecasting an Analytical Services, 2015) during peak times (AM and PM), indicating elevated traffic emissions, e.g. NO_*x*_ and VOCs. Traffic acceleration, flow and speed were not considered by this study, due to insufficient data at sampling sites, but differences between grouped traffic count data (although not correlated with NO_2_; Fig. 4) support site-specific traffic-related influences, such as number of cars, buses and duty vehicles, thus illustrating the importance of where NO_2_ measurements are undertaken and the need for high spatial resolution sampling (Beckwith et al., 2019).

Elevated NO_2_ concentrations, often > 40 µg m^−3^ EU/UK limit value, at diffusion tube locations (and automated monitoring stations, Fig. 2b) indicate deteriorated air quality by NO_2_ across Manchester. Due to varying sources, e.g. domestic heating, power generation and vehicular emissions, and relatively short lifetime of NO_2_, these concentrations are often strongly spatially variable (Cyrys et al., 2012; Lin et al., 2016; Weissert et al., 2018). Site-specific influences, e.g. from domestic combustion (DEFRA, 2017b), in more residential surroundings could explain recorded NO_2_ variability. For instance, public electricity and heat production accounted for approximately 20% of UK emissions in 2017 (NAEI, 2018b). However, NO_2_ concentrations at roadside locations and within the densely built-up city centre were generally higher than ‘background’ sites, e.g. deployment sites located further away from major roads in green spaces and residential areas.

Street geometry (e.g. height-to-width ratio and building arrangements), intersections and altered wind regimes (e.g. velocity and direction) all have major roles on NO_2_ distribution and dispersion in urban environments and human exposure (Fu et al., 2017; Kubota et al., 2008; Shen et al., 2017). For instance, Longley et al. (2004) highlighted street canyon and wind direction effects and subsequent dispersion of pollutants within Manchester’s city centre, which is comparable to results of this study. Albeit a reduced consideration of urban factors in this study, findings presented indicate problematic NO_2_ concentrations in Manchester city centre at locations not covered by automated air quality monitoring stations. Apparently urban layout effects (e.g. building heights and density), traffic regimes (e.g. traffic counts) and additional sources (e.g. domestic combustion) influenced dispersion and distribution of NO_2_ in Manchester (Fig. 4) that warrant further investigation, particularly in the context of urban air quality improvement plans in Manchester (TfGM, 2016).

### Temporal, including seasonal, variability of NO_2_ concentrations

**Fig. 5:**
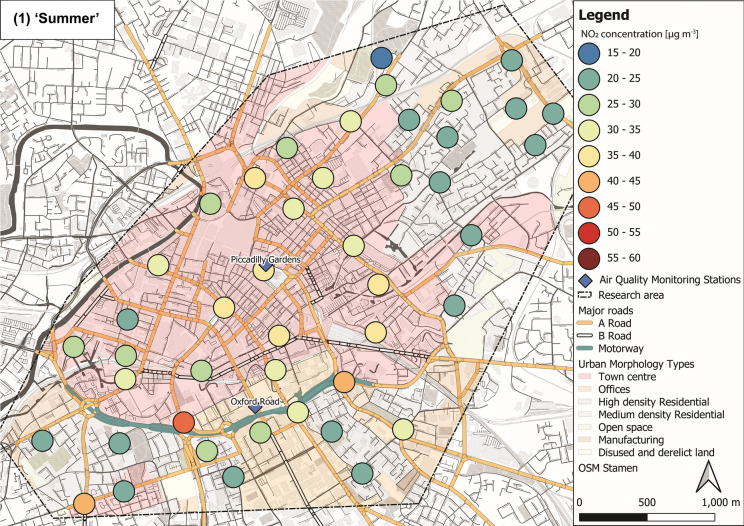

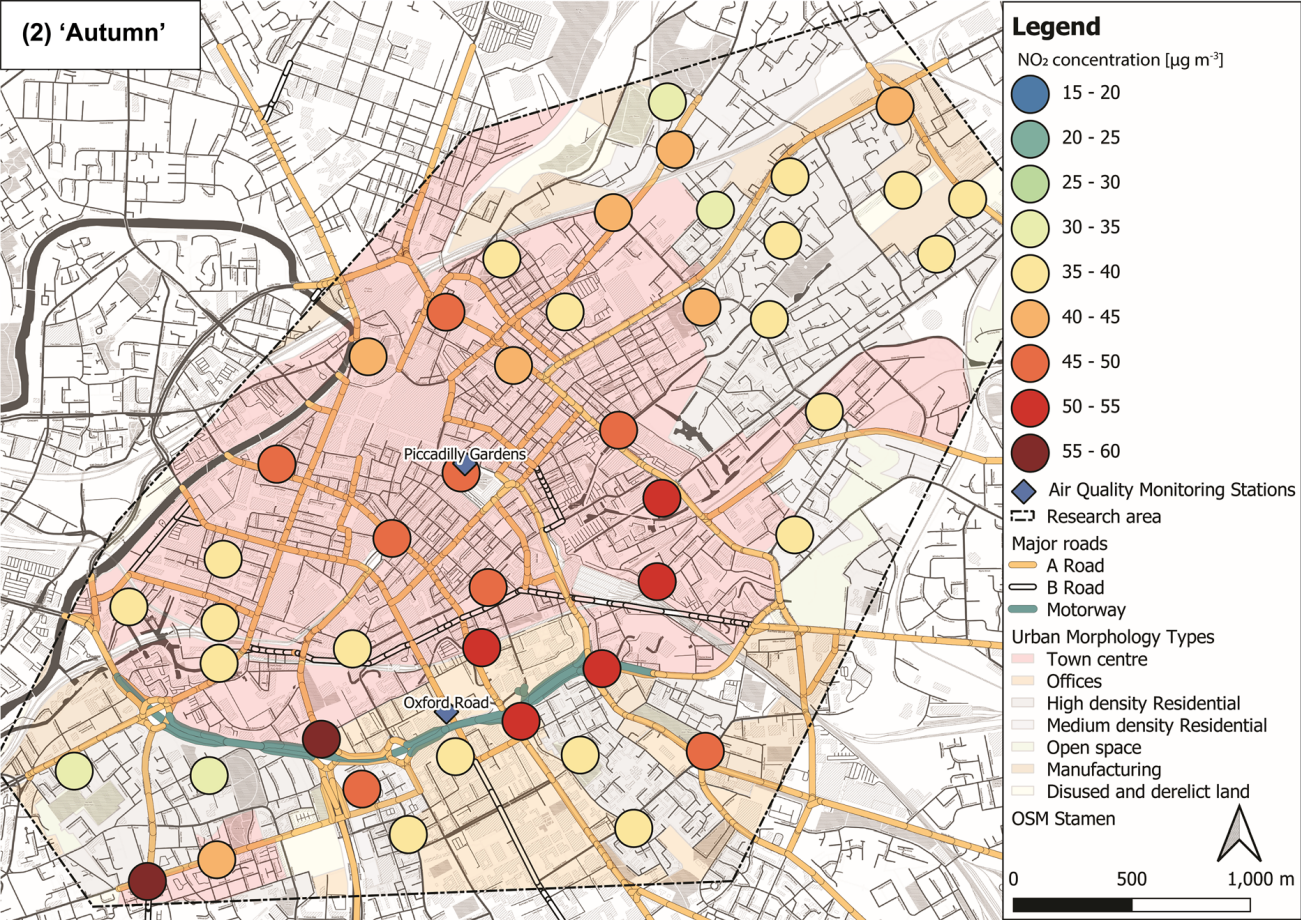

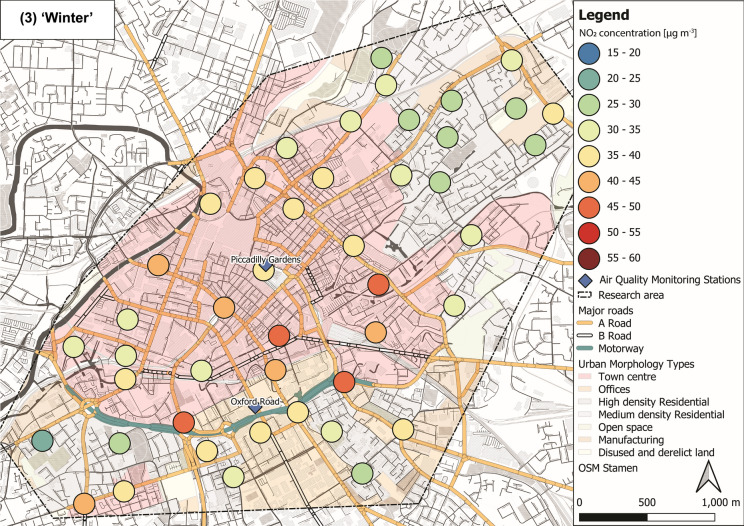

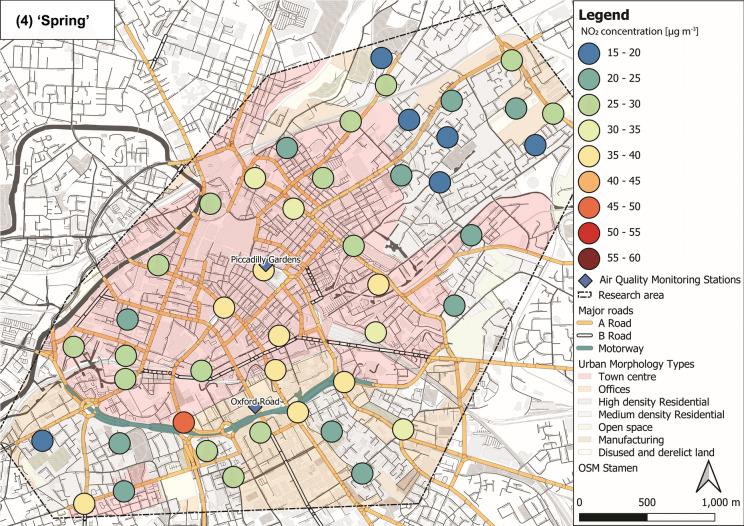
Maps of NO_2_ concentrations representing seasons: (1) ‘summer’ (July, August and September 2017), (2) ‘autumn’ (October, November and December 2017), (3) ‘winter’ (January, February and March 2018) and (4) ‘spring’ (April, May and June 2018), displayed with major roads and automated air quality monitoring stations

NO_2_ concentrations varied through the ‘seasons’ within the sampled Manchester urban area (Fig. 5). Lower mean NO_2_ levels were recorded during ‘summer’ (29 ± 7 µg m^−3^; Fig. 5(1)) and ‘spring’ (27 ± 7 µg m^−3^; Fig. 5(4)), whereas elevated concentrations were observed during ‘autumn’ (43 ± 7 µg m^−3^; Fig. 5(2)) and ‘winter’ (35 ± 6 µg m^−3^; Fig. 5(3); Tab. S6 ± 1*σ*). Throughout the 12-month deployment period, elevated NO_2_ (> 30 µg m^−3^) was recorded within the city centre area, whereas generally lower NO_2_ was recorded in more residential and open surroundings (i.e. north-east and south-west of the research area; Fig. 5). Overall, NO_2_ levels across Manchester decreased during warmer months compared to NO_2_ concentrations (> 30 µg m^−3^) during colder months (Fig. 5), due to photochemical processes (i.e. photolysis of NO_2_) in the presence of sunlight (Atkinson, 2000; Clapp & Jenkin, 2001). NO_*x*_ and VOCs (both from vehicular emissions) are key components in photochemical formation of ozone (O_3;_ Crutzen, 1995; National Research Council, 1991; Xue et al., 2013), which is also linked to severe human health impacts (Brunekreef & Holgate, 2002; Kampa & Castanas, 2008; WHO, 2013). Because of the chemical coupling of NO_x_ and O_3_ (and VOCs), a reduction in NO_2_ concentrations is accompanied by an increase in O_3_ levels (Atkinson, 2000; Clapp & Jenkin, 2001). Hence, O_3_ was most likely high, when NO_2_ was low (i.e. during warmer seasons) and passive O_3_ measurements could have provided additional information on deteriorated air quality; however, this was out of scope for this study.

Seasonality of NO_2_ concentrations has been reported in urban areas across the UK, including this study (Bower et al., 1991; Hewitt, 1991; Lin et al., 2016; Vardoulakis et al., 2011). Elevated ambient NO_2_ concentrations are usually ascribed to anthropogenic emissions and weather conditions, e.g. higher traffic counts, slower traffic movement and heating (Fantozzi et al., 2015; Matthaios et al., 2019). Furthermore, uptake of NO_2_ by tree leaves does not occur during winter (Desyana et al., 2017). In contrast, low NO_2_ concentrations are generally related to higher rainfall, increased solar radiation, higher temperatures, increased wind speeds and photolysis of NO_2_ (Caballero et al., 2012; Fantozzi et al., 2015; Heal et al., 2019; Vardoulakis et al., 2011). However, Kwak et al. (2017) reported higher NO_2_ during rainfall events, with regard to increased traffic volumes and slower traffic speed.

In this study, diffusion tubes were deployed on urban trees that can act to increase and decrease airborne pollutant concentrations, e.g. by enhanced deposition (air quality improvement) and/or impaired dispersion (air quality deterioration; Janhäll, 2015; Salmond et al., 2013; Weissert et al., 2018). Impacts by urban green (e.g. trees) during the diffusion tube deployment periods could not be considered to quantify effects on passive NO_2_ measurements. However, passively derived NO_2_ concentrations over 12 months showed deteriorated air quality for different seasons and over a wider area than regularly covered by automated measuring stations, which could inform local authorities about NO_2_ hotspots, which can be further evaluated (i.e. by using a more robust three-diffusion-tube approach) for mitigation strategies and to reduce human exposure.

### Exceedances of UK/EU regulatory NO_2_ limits of 40 µg m^−3^

**Fig. 6:**
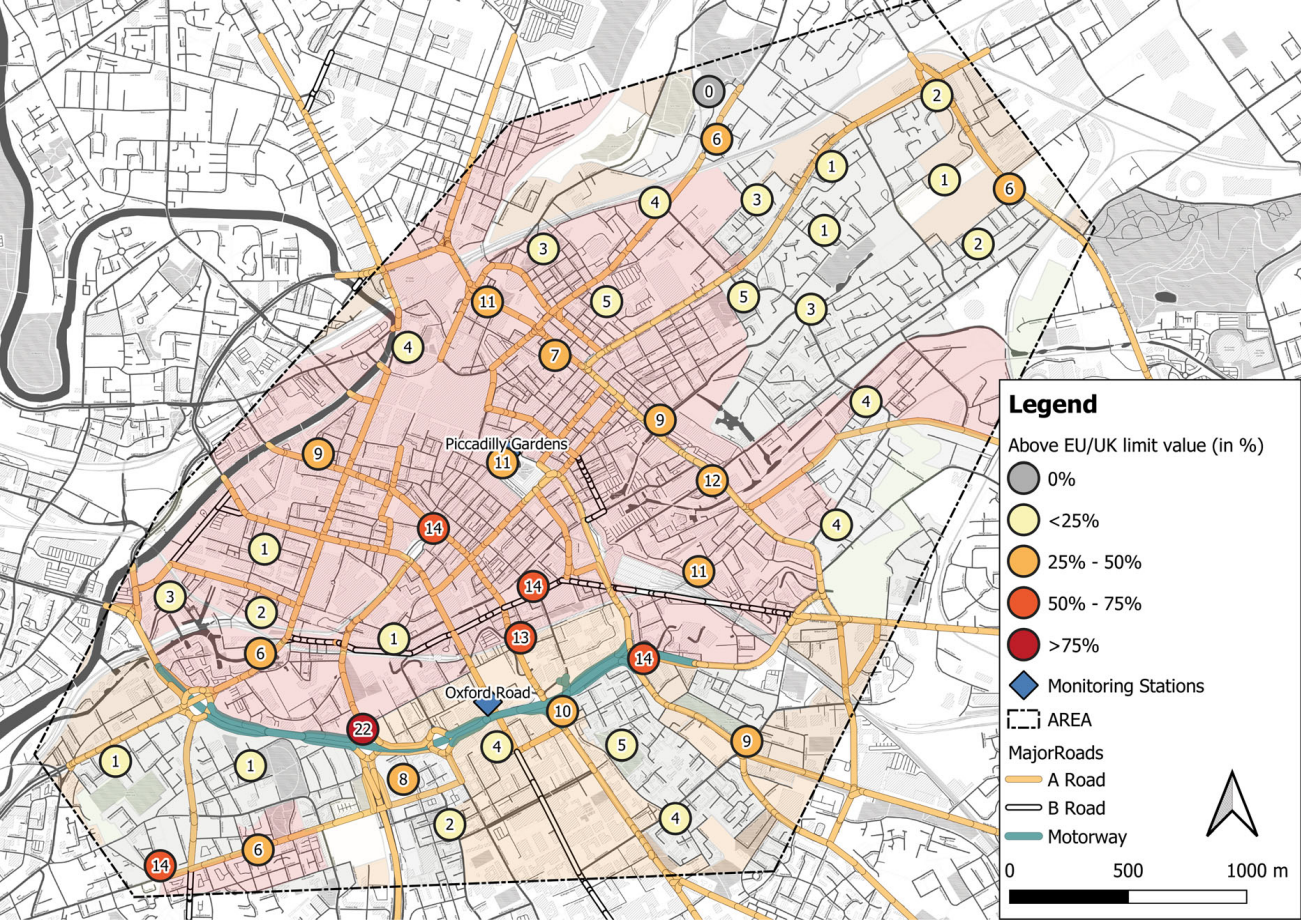
Diffusion tube deployment sites that exceeded the EU/UK limit value of 40 µg m^−3^, displayed with the number of exceedances out of 24 bi-weekly deployments (colour coded by percentage of exceedance); one site was recorded (grey circle) with no exceedance for the entire 12-month deployment period; urban morphology/land-use types as displayed in Fig. 1 are also shown to illustrate potential human health impacts across a wider area than the city centre, e.g. residential locations

Numerous exceedances of the EU/UK regulatory value (40 µg m^−3^; Fig. 6) were recorded by both automated monitoring stations and diffusion tube locations (Fig. S2b and Fig. 6). Exceedances of this regulatory value were recorded at 11 sites within the city centre area and along the major road network for the 12-month deployment period (Fig. 3, with ≥ 40 µg m^−3^; Fig. 6). Most notably, the road site location (ID: 30, ‘Mancunian Way’; Tab. S6; Fig. 6) exceeded the limit value 22 times out of the 24 successive deployments (annual average: 50 µg m^−3^). In contrast, only one location (ID: 26) did not exceed the limit throughout the 12-month deployment period (Tab. S6; Fig. 6), most likely because of its distance to road (150 m) and vicinity to green spaces.

It needs to be stated that bi-weekly passively derived NO_2_ concentrations were compared to an annual average regulatory value that is commonly used for continuously measured NO_2_ by automated monitoring stations. However, elevated passively derived NO_2_ (> 40 µg m^−3^) throughout the deployment period (2017–2018) suggests problematic NO_2_ across a wider area of Manchester city centre, e.g. north-east and south-west of city centre (Fig. 3, Fig. 6). A total of 16% (*N* = 7) of 45 sites exceeded the EU/UK regulatory value for at least 6 months, during this study’s period, suggesting long-term exposure to NO_2_ levels above the limit value (40 µg m^−3^, EU, 2008), posing a potential human health risk. Nonetheless, toxic effects of cumulative and/or chronic NO_2_ exposure may have adverse health effects at lower concentration levels for local populations (COMEAP, 2018).

NO_2_ concentrations have declined since continuous measurements of NO_2_ commenced in Manchester (in 1987; Fig. S2a), because of technical improvements (i.e. introduction of EURO emission standards; Fig. S2a) and reduced emissions from transport (NAEI, 2018a). However, real-world emissions of diesel vehicles, with the newest EURO 5 and EURO 6 standards, have been up to 20 times higher than the allowed emission levels (Barrett et al., 2017; European Environment Agency, 2018). It can therefore be assumed that long-term exposure to elevated NO_2_ concentrations across Manchester city centre contributes to overall poor human health statistics. For instance, reducing NO_2_ concentrations in Manchester by 5 µg m^−3^ could prevent 160 premature deaths and reduce the amount of days spent in hospital by 350 days per year (CBI Economics, 2021). Moreover, Achakulwisut et al. (2019) reported that 19% of childhood asthma within the UK is related to air pollution, particularly NO_2_. Incorporating additional pollutants, e.g. NO, O_3_, VOCs and PM_10_ (Clapp & Jenkin, 2001; Vardoulakis et al., 2011), in future passive sampling studies could improve further spatial air quality and health impact assessments in highly populated urban environments (Pannullo et al., 2015). Frequent exceedances, as reported for this study, imply a considerable impact on human health across the wider study area and importance of ameliorating NO_2_ concentrations in Manchester.

This study has provided an overview of EU/UK regulatory value exceedances across Manchester. Local authorities, e.g. Manchester City Council, could use such information on exceedances of regulatory limits to benefit public engagement during high pollution events (i.e. seasonal degradation of air quality) and towards improvements of identified hotspot areas in order to reduce high NO_2_ concentrations. Such action is particularly necessary at major roads frequently used for personal and public transport. However, more open less densely built-up areas also showed elevated NO_2_, indicating the necessity for additional measurements to support reduction of NO_2_ across Manchester and thereby reduce long-term health impacts for local population.

## Conclusion

This study has assessed spatial variability of NO_2_ concentrations using a single-NO_*x*_ diffusion tube network across an urban environment, to supplement automated air quality monitoring stations, to investigate whether air quality targets (i.e. EU/UK regulatory value of 40 µg m^−3^) can be met and identify areas that may pose a risk to human health.

A considerable NO_2_ pollution problem across a wide area of Manchester was evidenced, illustrating the key challenge to reduce NO_2_ levels and subsequently human exposure. EU/UK regulatory value (40 µg m^−3^) exceedances were recorded for 11 out of 45 sites (annual average NO_2_ concentrations), particularly at locations that are not continuously monitored by automated air quality measurement stations. These results could be of importance when assessing NO_2_ exposure and health impacts on Manchester’s urban population, particularly when coinciding with episodes of elevated NO_2_ pollution. For instance, concentrations of NO_2_ were the highest during cold periods, whereas low NO_2_ was recorded during warm episodes. Concentrations of O_3_ were most likely to be higher when NO_2_ was low, which is also linked to severe human health impacts (Kampa & Castanas, 2008). Consequently, additional measurements of pollutants closely linked with NO_2_ (i.e. NO, O_3,_ VOCs and PM_10_; (Clapp & Jenkin, 2001; Fantozzi et al., 2015; Vardoulakis et al., 2011) could be included to evaluate deteriorated air quality and estimate public health impacts in more detail. Therefore, high spatial coverage of NO_2_ pollution can support local authorities to inform placement of additional monitoring locations within air quality management areas (AQMAS) and evaluate effectiveness of pollutant reduction programmes.

Although only a single diffusion tube was deployed, the easy-to-use and cost-effective approach has provided information about deteriorated air quality in the urban environment of Manchester. Such an approach could be applied as an initial screening tool in a comparable urban environment, particularly where resources (i.e. financial, personnel and equipment) are limited. Furthermore, it can be applied to support additional air quality monitoring campaigns, e.g. a biomonitoring approach. However, carefully conducted tube preparation, extraction and co-location with automated measurements are important considerations to evaluate the validity of measurements. Indeed, identified hotspots of NO_2_ pollution should be extended further using the recommended three-diffusion-tube approach, together with fine spatial detail of the sampling locations surrounding, to provide additional insights into pollutant distribution, dispersion and human exposure across urban environments.

## Supplementary Information

Below is the link to the electronic supplementary material.

Supplementary file1 (DOCX 3392 KB)

## Data Availability

Data are available within the article or its supplementary materials.
