# Supplementary material for: Spatiotemporal variability of nitrogen dioxide (NO2) pollution in Manchester (UK) city centre (2017–2018) using a fine spatial scale single-NOx diffusion tube network
Source: Environ Geochem Health. 2021 Nov 5;44(11):3907–27. doi: 10.1007/s10653-021-01149-w (PMC9587101; doi:10.1007/s10653-021-01149-w)
Supplement: Supplementary file 1 — Supplementary file1 (DOCX 3392 KB) [file 10653_2021_1149_MOESM1_ESM.docx]

# **Spatiotemporal variability of nitrogen dioxide (NO_2_) pollution in Manchester (UK) city centre (2017-2018) using a fine spatial scale single-NO_x_ diffusion tube network**

Daniel Niepsch^1^, Leon J. Clarke^1^, Konstantinos Tzoulas^1^ and Gina Cavan^1^

^1^ Department of Natural Sciences, Faculty of Science and Engineering, Manchester Metropolitan University, Chester Street, Manchester, M1 5GD, UK

Corresponding author: [Daniel.Niepsch@gmx.de](mailto:Daniel.Niepsch@gmx.de)

**OrcID**

Daniel Niepsch - <https://orcid.org/0000-0002-0177-1996>

# **Supplementary Material**

**Table S1:** Diffusion tube locations, by ID (as shown in Figure 1) and XY coordinates (OSGB 1936/British National Grid) together with nitrogen contents (N wt%; analysed by LECO CN analyser; Niepsch, 2019) in *Xanthoria parietina* to inform diffusion tube locations to measure atmospheric NO_2_ [µg m^-3^]; lichen N wt% can reflect airborne nitrogen loads, including from anthropogenic impacts (Boltersdorf et al. 2014) and elevated N wt% in lichens could indicate elevated nitrogen pollution.

| **ID** | **XY coordinates** | **N wt%** | **ID** | **XY coordinates** | **N wt%** | **ID** | **XY coordinates** | **N wt%** |
| --- | --- | --- | --- | --- | --- | --- | --- | --- |
| **1** | X: 385143  Y: 398190 | 2.50 | **16** | X: 383902  Y: 398788 | 3.07 | **31** | X: 383263  Y: 396662 | 2.05 |
| **2** | X: 384734  Y: 398978 | 3.59 | **17** | X: 384382  Y: 397558 | 3.18 | **32** | X: 382970  Y: 396570 | 3.28 |
| **3** | X: 384231  Y: 398977 | 3.18 | **18** | X: 384295  Y: 398297 | 2.95 | **33** | X: 382661  Y: 397036 | 3.07 |
| **4** | X: 384467  Y: 399200 | 2.18 | **19** | X: 384003  Y: 398020 | 3.03 | **34** | X: 384271  Y: 397097 | 3.05 |
| **5** | X: 384960  Y: 398477 | 2.68 | **20** | X: 382892  Y: 397730 | 2.02 | **35** | X: 385312  Y: 398998 | 2.21 |
| **6** | X: 384890  Y: 397470 | 2.61 | **21** | X: 383290  Y: 397932 | 2.71 | **36** | X: 385653  Y: 399279 | 2.44 |
| **7** | X: 385326  Y: 397120 | 2.56 | **22** | X: 383273  Y: 397492 | 2.81 | **37** | X: 386160  Y: 399492 | 2.88 |
| **8** | X: 385024  Y: 396794 | 2.48 | **23** | X: 383277  Y: 397664 | 3.41 | **38** | X: 386128  Y: 399848 | 2.70 |
| **9** | X: 384548  Y: 397247 | 3.06 | **24** | X: 384516  Y: 398751 | 1.96 | **39** | X: 385684  Y: 399549 | 2.55 |
| **10** | X: 384798  Y: 397104 | 2.42 | **25** | X: 384409  Y: 397812 | 3.55 | **40** | X: 384938  Y: 399397 | 3.03 |
| **11** | X: 385829  Y: 398555 | 1.01 | **26** | X: 385166  Y: 399865 | 2.45 | **41** | X: 385369  Y: 399408 | 2.78 |
| **12** | X: 385704  Y: 398035 | 3.60 | **27** | X: 383876  Y: 396960 | 3.02 | **42** | X: 385199  Y: 399664 | 3.02 |
| **13** | X: 385123  Y: 397837 | 3.04 | **28** | X: 384072  Y: 396768 | 1.84 | **43** | X: 386434  Y: 399454 | 3.04 |
| **14** | X: 383836  Y: 397553 | 3.77 | **29** | X: 383231  Y: 397017 | 2.20 | **44** | X: 386302  Y: 399221 | 2.86 |
| **15** | X: 383517  Y: 398334 | 2.42 | **30** | X: 383705  Y: 397171 | 3.35 | **45** | X: 385597  Y: 398946 | 2.47 |

**Table S2:** Actual diffusion tube deployment periods over a 12-month interval (start on 3^rd^ July 2017 to end on 28^th^ June 2018; 361 days)

| **Deployment periods for NO_x_ diffusion tubes** | |
| --- | --- |
| **(1)** 03/07/2017 to 20/07/2017 | **(13)** 11/01/2018 to 25/01/2018 |
| **(2)** 20/07/2017 to 03/08/2017 | **(14)** 25/01/2018 to 08/02/2018 |
| **(3)** 03/08/2017 to 24/08/2018 – 21 days (conference attendance) | **(15)** 08/02/2018 to 22/02/2018 |
| **(4)** 24/08/2017 to 07/09/2017 | **(16)** 22/02/2018 to 08/03/2018 |
| **(5)** 07/09/2017 to 21/09/2017 | **(17)** 08/03/2018 to 22/03/2018 |
| **(6)** 21/09/2017 to 05/10/2017 | **(18)** 22/03/2018 to 05/04/2018 |
| **(7)** 05/10/2017 to 19/10/2017 | **(19)** 05/04/2018 to 19/04/2018 |
| **(8)** 19/10/2017 to 02/11/2017 | **(20)** 19/04/2018 to 03/05/2018 |
| **(9)** 02/11/2017 to 16/11/2017 | **(21)** 03/05/2018 to 17/05/2018 |
| **(10)** 16/11/2017 to 30/11/2017 | **(22)** 17/05/2018 to 31/05/2018 |
| **(11)** 30/11/2017 to 14/12/2017 | **(23)** 31/05/2018 to 14/06/2018 |
| **(12)** 14/12/2017 to 11/01/2018 – 28 days (Christmas/winter break) | **(24)** 14/08/2018 to 28/06/2018 |

**Table S3:** Meteorological data (obtained from the Whitworth Meteorological Observatory; “Whitworth Meterological Observatory - Data Archive,” 2018) for bi-weekly deployment periods* (dates stated and numbered in Table S2); N/A – no data available for bi-weekly deployment.

| **Deployment period*** | **Min Temp (°C)** | **Max Temp (°C)** | **Mean Temp (°C)** | **Mean relative humidity (%)** | **Mean wind speed (m/s)** | **Mean wind direction (°)** | **Total precipitation (mm)** | **Total sunshine hours (h)** |
| --- | --- | --- | --- | --- | --- | --- | --- | --- |
| (1) | 11.00 | 26.00 | 17.07 | N/A | 2.38 | 217.72 | 25.69 | 65.17 |
| (2) | 10.60 | 22.20 | 16.10 | N/A | 3.32 | 216.26 | 69.91 | 32.89 |
| (3) | 9.40 | 26.60 | 15.81 | N/A | 3.13 | 202.24 | 56.72 | 63.08 |
| (4) | 8.60 | 22.90 | 15.27 | N/A | 2.55 | 216.13 | 25.42 | 14.04 |
| (5) | 7.30 | 19.00 | 13.22 | N/A | 2.94 | 241.90 | 69.12 | 36.67 |
| (6) | 8.20 | 20.80 | 14.36 | N/A | 3.38 | 193.06 | 54.75 | 1.05 |
| (7) | 7.80 | 20.60 | 14.25 | N/A | 4.06 | 217.95 | 34.16 | 15.71 |
| (8) | 2.20 | 18.10 | 11.70 | N/A | 2.99 | 224.85 | 50.43 | 8.66 |
| (9) | 0.00 | 13.30 | 8.69 | N/A | 2.14 | 237.62 | 30.93 | 13.34 |
| (10) | 0.00 | 15.50 | 7.05 | N/A | 3.28 | 254.14 | 55.37 | 7.35 |
| (11) | -0.68 | 11.86 | 5.68 | 94.81 | 3.03 | 252.54 | 56.88 | 6.07 |
| (12) | -1.54 | 13.28 | 5.48 | 92.67 | 3.27 | 209.23 | 166.39 | 16.58 |
| (13) | -1.54 | 13.28 | 5.17 | 92.80 | 4.03 | 207.66 | 80.38 | 1.07 |
| (14) | -2.58 | 13.39 | 4.73 | 89.73 | 3.16 | 222.24 | 32.15 | 20.93 |
| (15) | -0.38 | 10.80 | 5.06 | 87.61 | 3.50 | 206.41 | 43.02 | 21.03 |
| (16) | -5.24 | 9.48 | 1.83 | 81.01 | 3.71 | 133.78 | 35.63 | 33.36 |
| (17) | -2.42 | 14.61 | 5.66 | 84.01 | 3.51 | 154.65 | 59.21 | 27.81 |
| (18) | 0.00 | 14.69 | 6.78 | 82.26 | 2.59 | 187.31 | 47.16 | 32.66 |
| (19) | 0.00 | 24.38 | 10.46 | 81.16 | 2.80 | 173.73 | 22.34 | 39.01 |
| (20) | 0.00 | 24.38 | 10.46 | 73.18 | 3.27 | 206.57 | 32.18 | 59.85 |
| (21) | 0.00 | 26.37 | 13.38 | 68.92 | 2.18 | 214.83 | 8.02 | 110.08 |
| (22) | 0.00 | 25.64 | 15.69 | 68.79 | 2.57 | 102.79 | 11.69 | 113.32 |
| (23) | 7.27 | 25.67 | 16.62 | 73.27 | 2.15 | 148.82 | 8.86 | 85.59 |
| (24) | 9.63 | 30.47 | 16.91 | 63.64 | 3.04 | 224.71 | 4.94 | 111.16 |

**
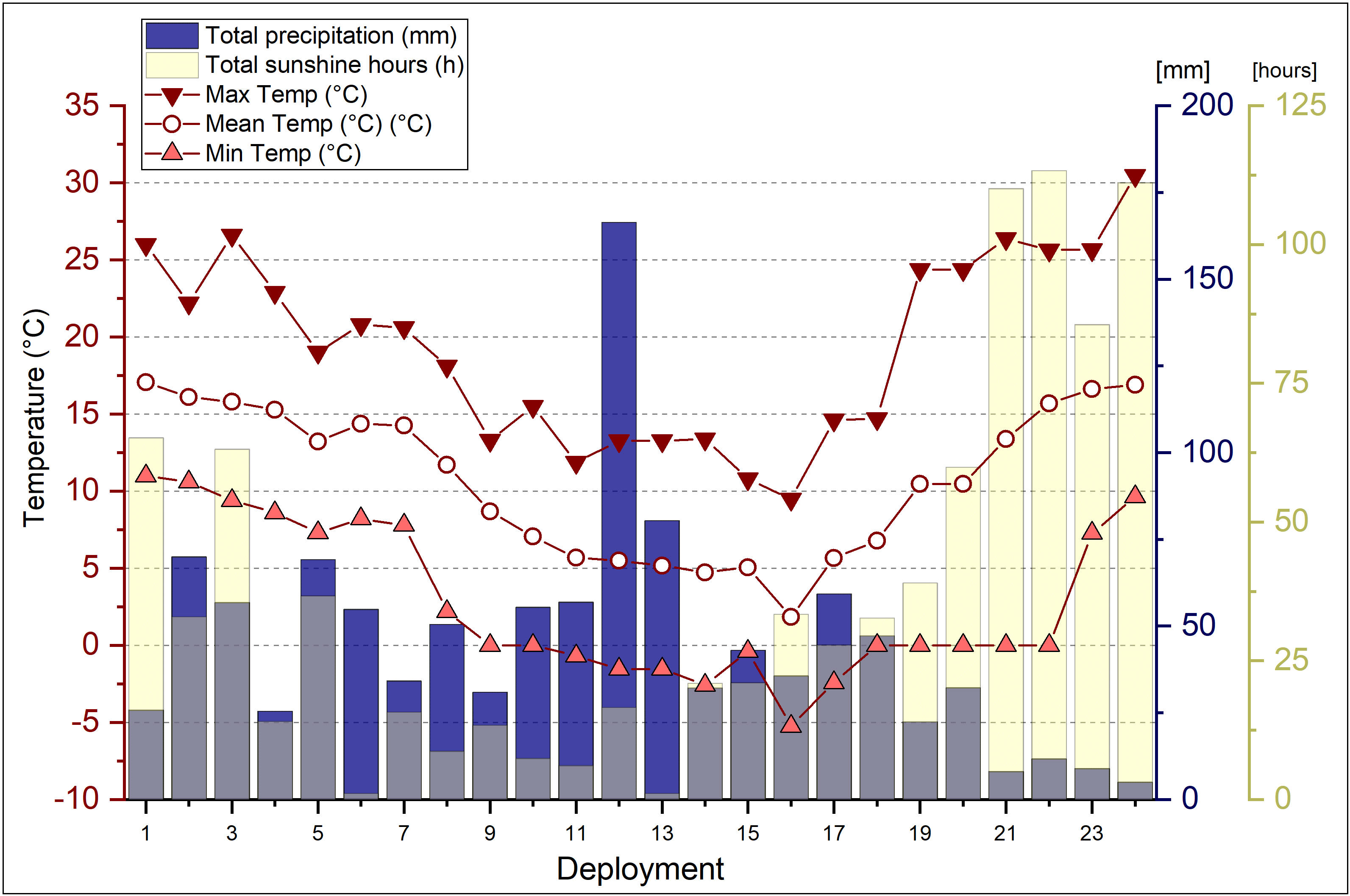
**

**
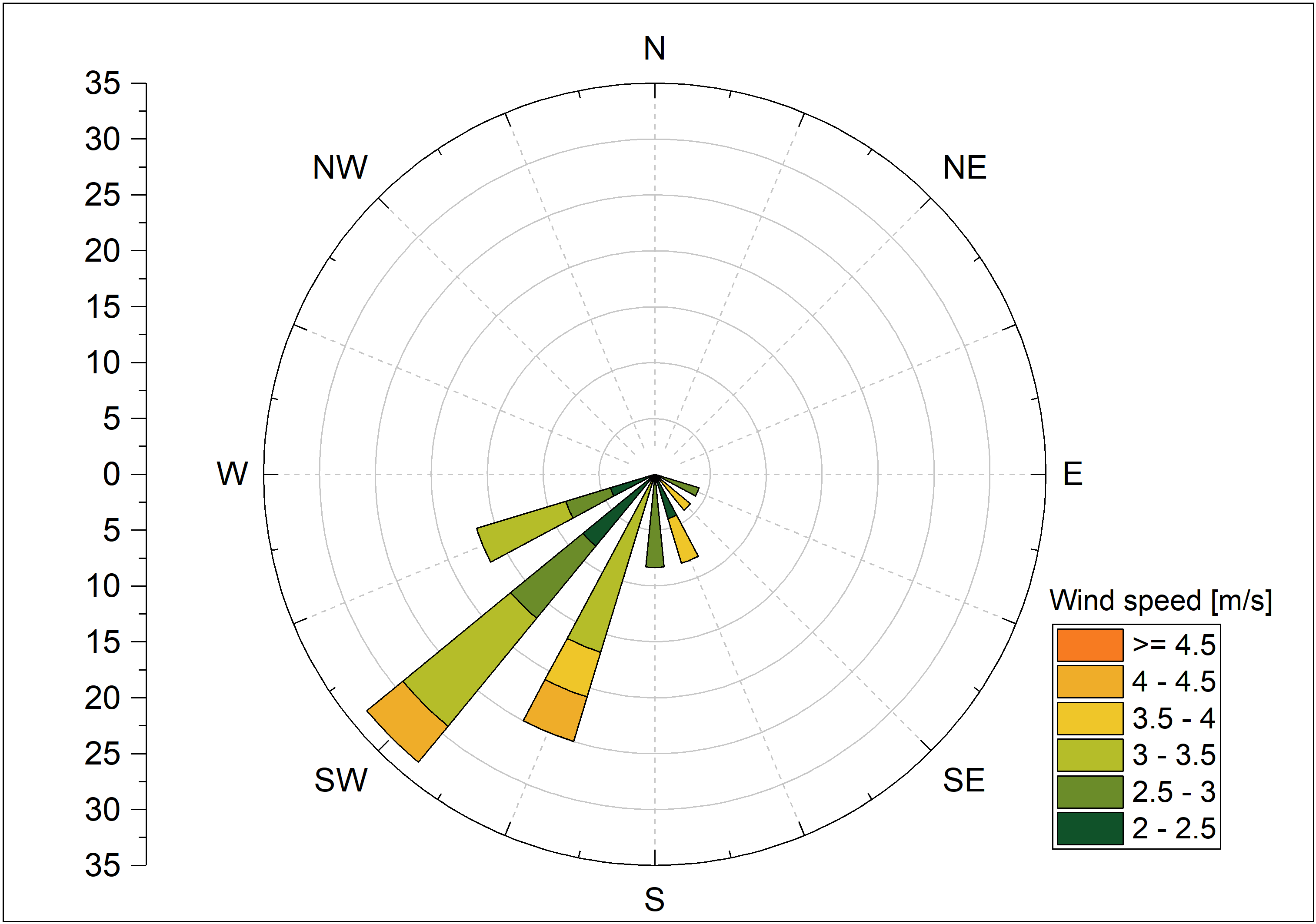
**

**Figure S1:** Temperature (minimum, maximum and mean; °C), total precipitation (mm), total sunshine (hours) [upper panel] and mean wind direction (frequency) and wind speed (m/s) during the 24 bi-weekly NO_x_ diffusion tube deployments


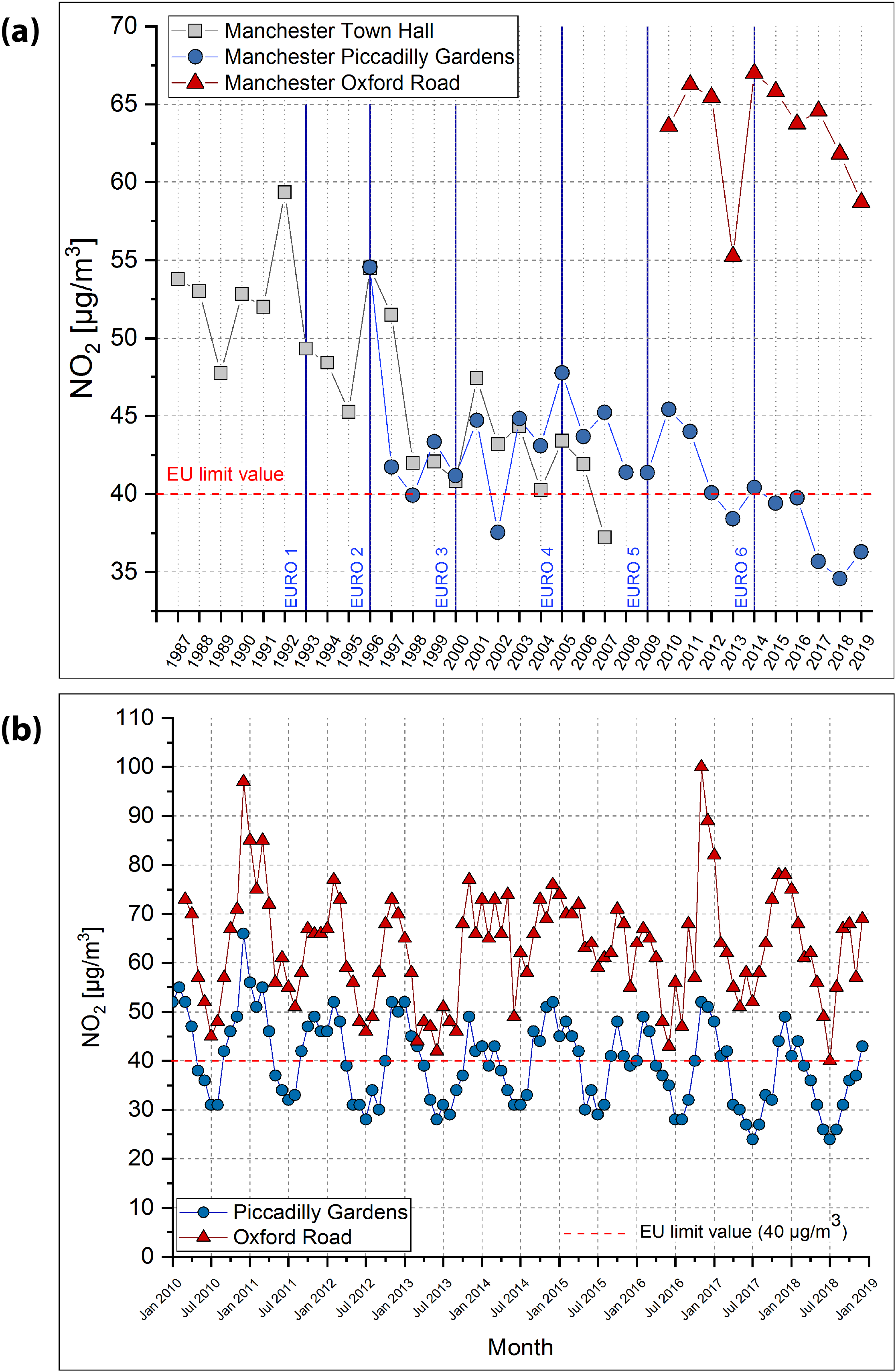


**Figure S2:** (a) NO_2_ concentrations [µg m^-3^] annual mean between 1987 and 2019 measured at Manchester automated monitoring stations (including Town Hall monitoring station – terminated in 2007), with introduction dates of EURO emission standards and (b) monthly mean at the automated monitoring stations (Piccadilly Gardens: blue and Oxford Road: red) for the years 2010 to 2019 (Air Quality England, 2018a, 2018c). This figure illustrates the problem of NO_2_ measured by only two automated monitoring stations across the City of Manchester for the last decade which is particularly evident for Manchester Oxford Road.

**Table S4:** Urban influencing factors, data source and data classification justification, based on pollutant dispersion and human health studies for high-spatial resolution analysis of air quality in Manchester, with utilised datasets and data sources, classification and justification of data classification

| Urban influencing factor | Dataset used and source | Data classification | Justification |
| --- | --- | --- | --- |
| Major road classes | Major road classes – A-, B-roads and motorways (Digimap - Ordnance Survey 2016) | **M** – motorway  **A** – A-Road  **B** – B-road  **[U** – unclassified] | Decline of NO_x_ and NO_2_ from major roads within the first 200 m (Bermejo-Orduna et al. 2014; Gilbert et al. 2003; Laffray et al. 2010) |
| Distance to major road | Categorised by GIS linear buffer (25, 50, 100 and 200m) superimposed on road network | **1:** <25 m  **2:** 25 to 50m  **3:** 50 to 100m  **4:** 100 to 200 m  **5:** >200m |  |
| Traffic counts | Annual Average Daily Traffic flow (AADF, 2017) – all vehicles (DfT 2017) | **1:** <10.000  **2:** 10.000 to 20.000  **3:** 20.000 to 30.000  **4:** >30.000 | Dynamic traffic movement from measured/estimated data – available as point data only; total vehicles per day used |
| Building heights | OS Building Heights, ‘relative height from ground level to highest part of rood (relHmax)’ (Digimap - Ordnance Survey 2017); mean building height in GIS 50m buffer of sampling site | **1:** <10m  **2:** 10 to 20m  **3:** >20m | High urban density affect flow patterns (i.e. ventilation), causing poor air and impacting on pedestrian health (Britter and Hanna 2003; Buccolieri et al. 2010; Hertel and Goodsite 2009; Lo and Ngan 2015; Shen et al. 2017) |


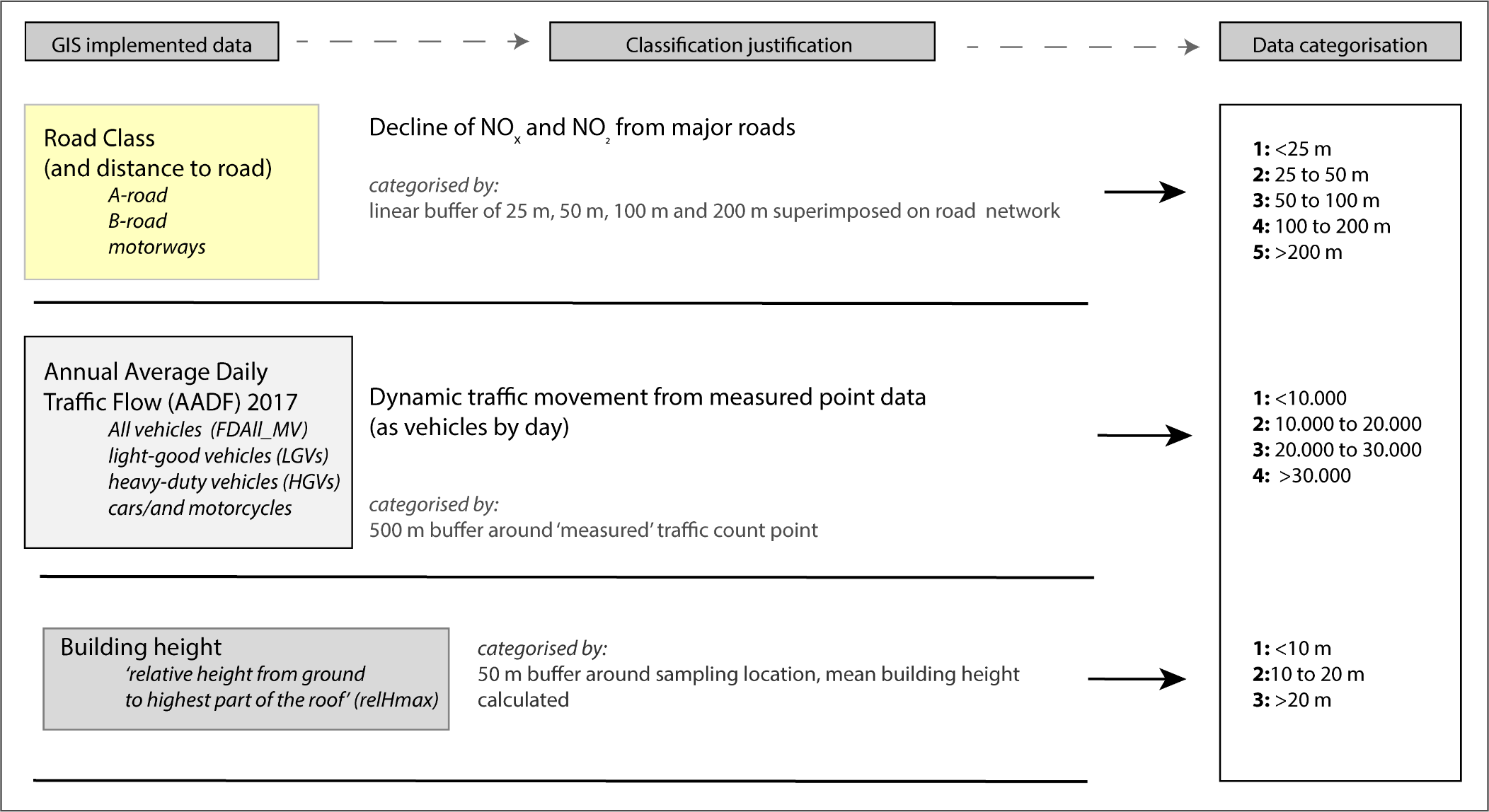


**Figure S3:** Schematic overview of categorisation of datasets for spatial analysis (as described in Table S-4) of NO­_2_ concentrations; urban influences were implemented into GIS software and sampling sites were categorised/grouped based on literature-informed pollutant declines (i.e. NO_2_) and human health studies; distances were measured from the specific sampling location

**Table S5:** Parameters used to calculate NO_2_ [µg/m^3^] derived from ion chromatography (IC) analysis of extracted NO_x_ diffusion tubes for nitrite (NO_2_^-^) following DEFRA guidelines for NO_2_ (Defra 2008; Massman 1998)

| Parameter | NO_2_ |
| --- | --- |
| Diffusion coefficient (D) in air for the UK | 1.46x10^-5^ m^2^/s  (at 11°C) **^#1^** |
| NO_x_ diffusion tube dimensions   - tube cross sectional area (a) - tube length (l) | 9.503 x10^-5^ m^2^  7.1 cm |
| Calculated ‘sampling rate’ for NO_x_ diffusion tubes deployed in Manchester | 70.3x10^-6^ m^3^/h |
| EU temperature correction (at 20°C; 293K) for comparability | 0.969 **^#2^** |
| ^#1:^ typical mean UK ambient temperature at 284K (11°C) (Defra 2008)  ^#2:^ correction from 284K to 293K (Defra 2008) | |

Calculations for NO_2_ concentrations in air followed the equations:

$C_{NO_{2}}= \frac{1}{'sampling rate^{'}}* \frac{m}{t}$ (1)

$'sampling rate^{'}= \frac{D_{NO_{2}}*a}{l}$ (2)

$C_{NO_{2}} =0.969* 14245* \frac{m}{t}$ (3)

with *C* being the concentration of NO_2_ in the atmosphere (µg/m^3^), *m* as the mass of nitrite (NO­_2_^-^) in diffusion tube (µg) and *t* as exposure time (hours). The sampling rate (2) is calculated by using the diffusion coefficient of NO_2_ through air (D), the cross sectional area (a) and the length of the diffusion tube (l). Parameters to calculate sampling rates for NO_2_ concentrations are displayed in **Table S5**, resulting in Eq. (3).


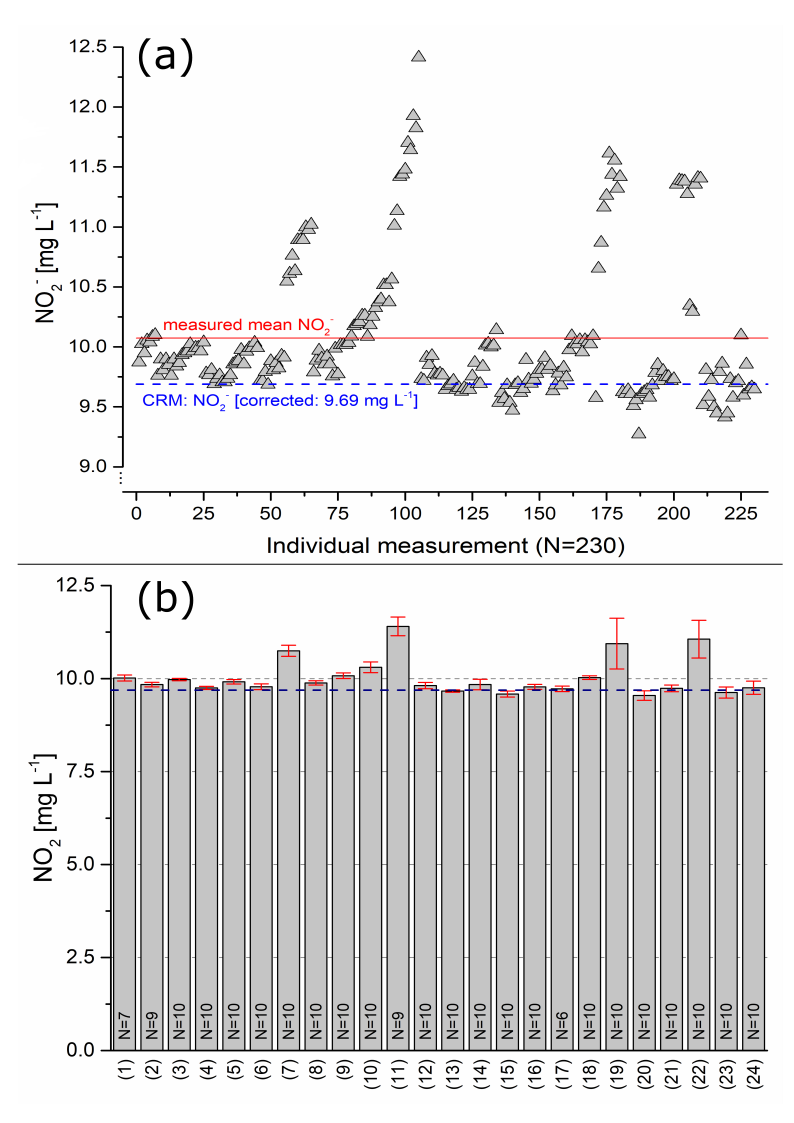


**Figure S4:**  (a) Analysed nitrite concentrations (N=230) in certified reference material (CRM Simple Nutrients – Whole Volume QC3198, Lot: LRAB1802^;^ displayed with certified value – corrected to NO_2_^-^ from 2.95 mg L^-1^ NO_2_-N, and measured mean by IC) and (b) mean nitrite concentrations (in CRM) by batch (blue dashed line illustrates the corrected certified value 9.96 mg L^-1^ NO_2_^-^; error bars presented as 1x standard deviation of repeated CRM measurements, displayed as N, for each analytical batch)


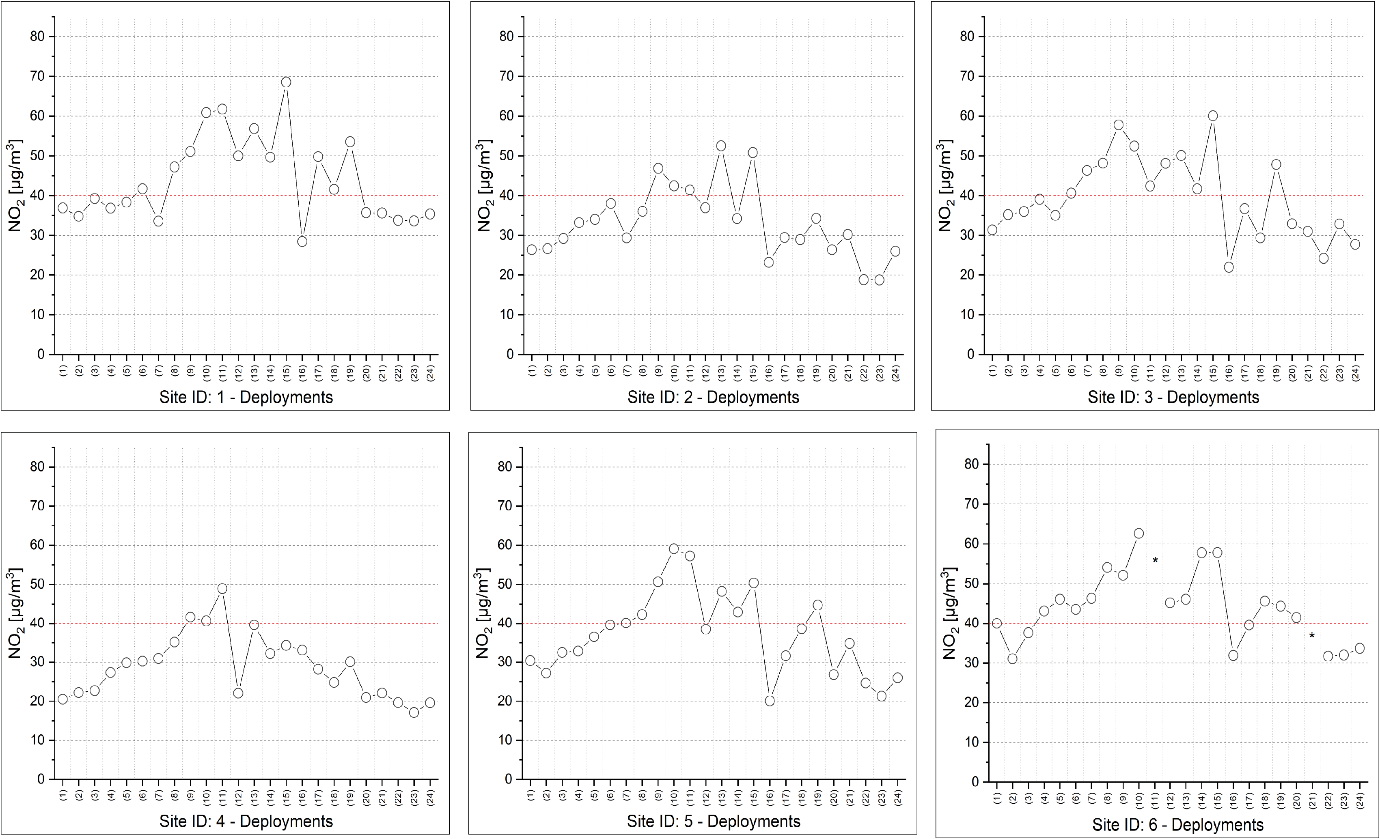


**Figure S5:** NO_2_ concentrations [µg m^-3^] recorded at sampling sites ID: 1 to ID: 6 for the deployment periods (1) to (24) [12-months, bi-weekly changes]; *indicates missing equipment/tube on ground (not analysed); EU/UK limit value of 40 µg/m^3^ (red dotted line) is also shown


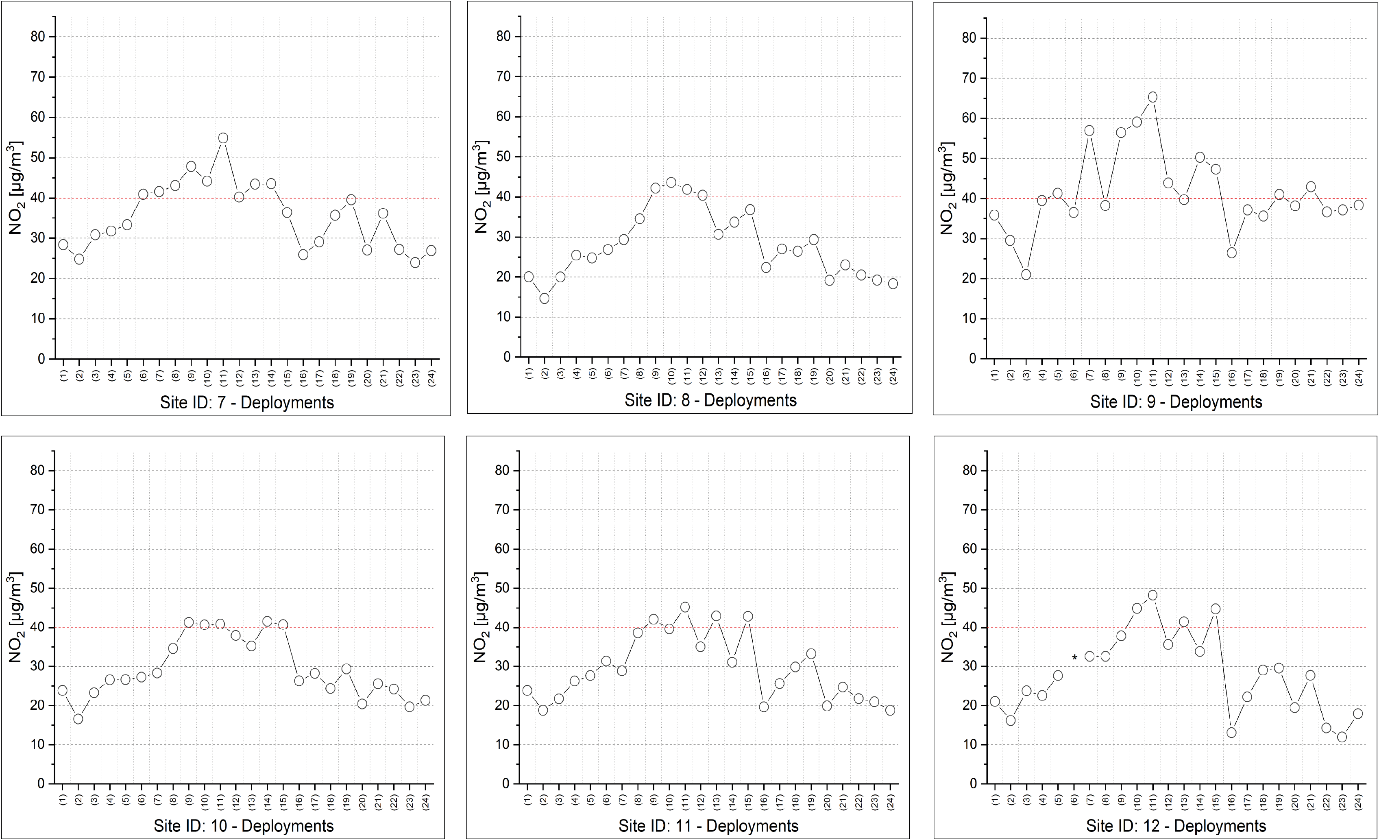


**Figure S6:** NO_2_ concentrations [µg m^-3^] recorded at sampling sites ID: 7 to ID: 12 for the deployment periods (1) to (24) [12-months, bi-weekly changes]; *indicates missing equipment/tube on ground (not analysed); EU/UK limit value of 40 µg/m^3^ (red dotted line) is also shown


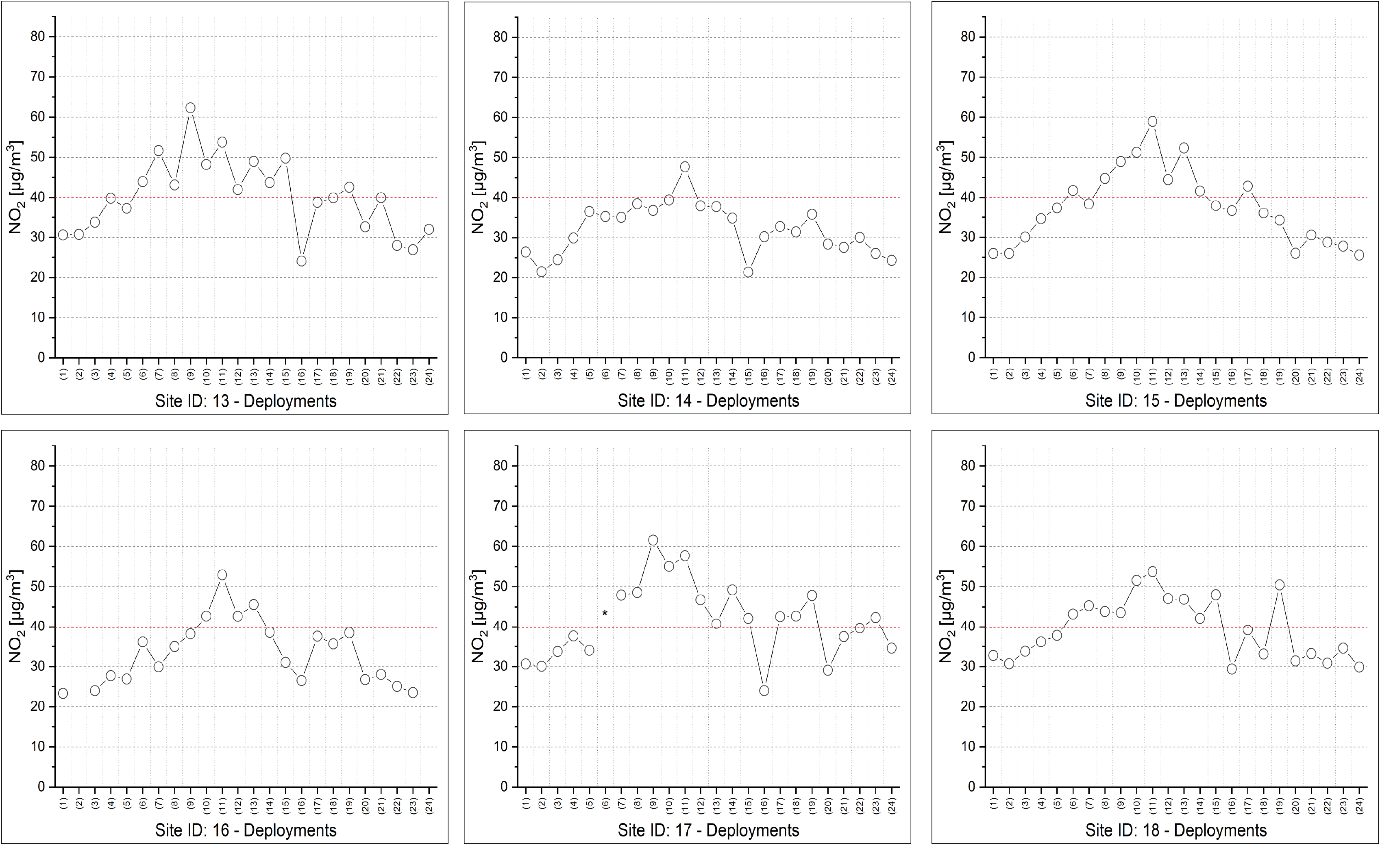


**Figure S7:** NO_2_ concentrations [µg m^-3^] recorded at sampling sites ID: 13 to ID: 18 for the deployment periods (1) to (24) [12-months, bi-weekly changes]; *indicates missing equipment/tube on ground (not analysed); EU/UK limit value of 40 µg/m^3^ (red dotted line) is also shown


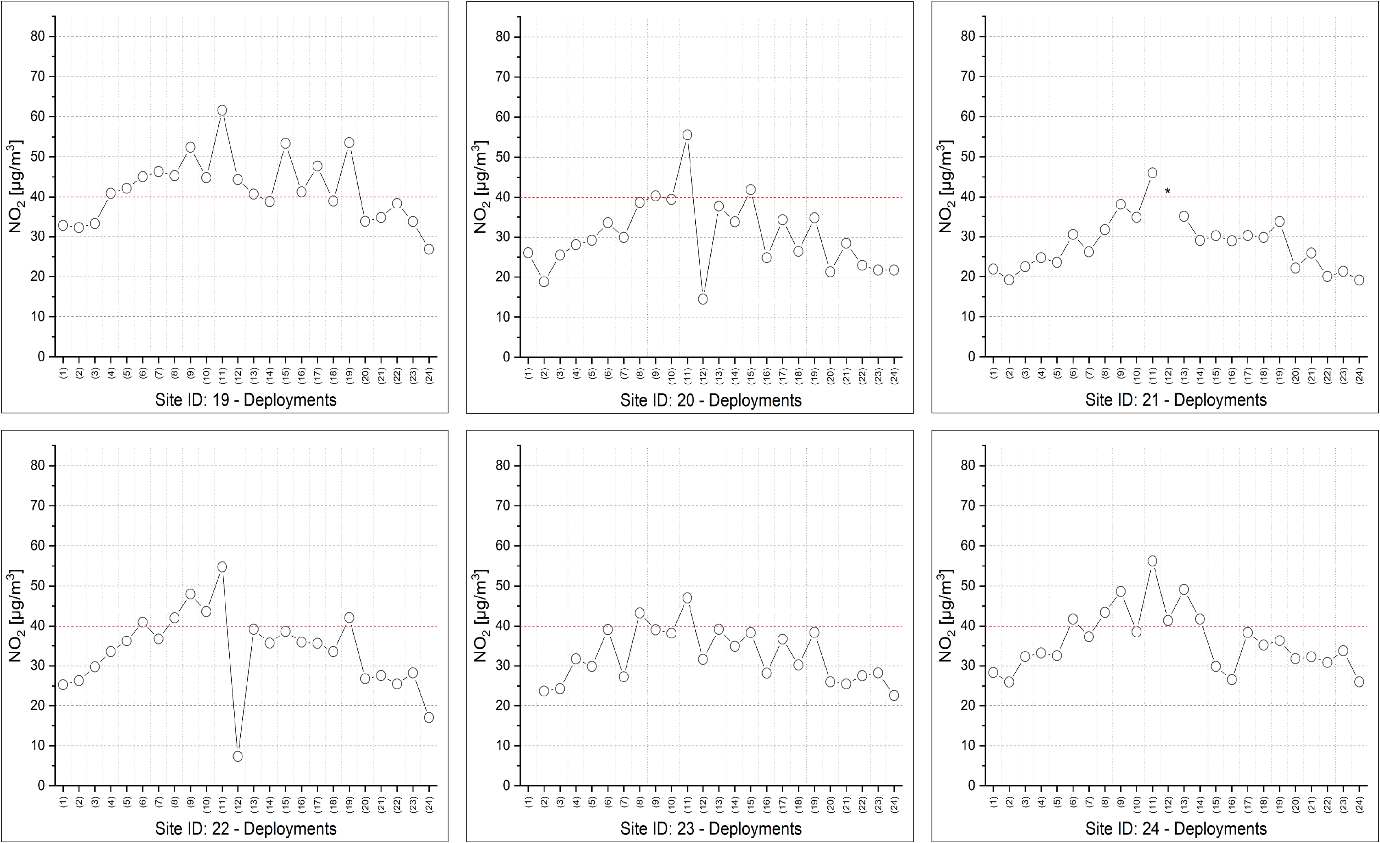


**Figure S8:** NO_2_ concentrations [µg m^-3^] recorded at sampling sites ID: 19 to ID: 24 for the deployment periods (1) to (24) [12-months, bi-weekly changes]; *indicates missing equipment/tube on ground (not analysed); EU/UK limit value of 40 µg/m^3^ (red dotted line) is also shown


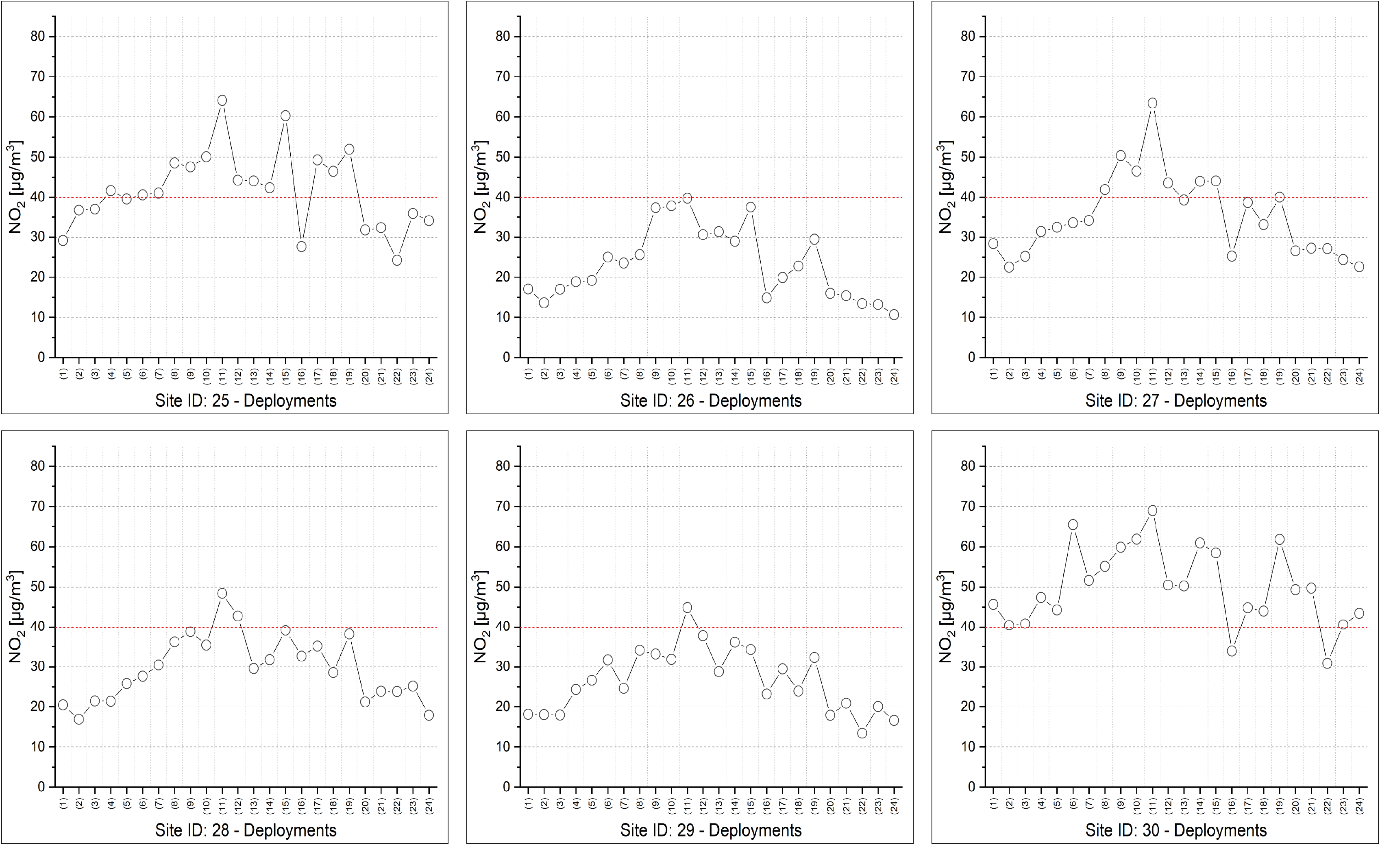


**Figure S9:** NO_2_ concentrations [µgm^-3^] recorded at sampling sites ID: 25 to ID: 30 for the deployment periods (1) to (24) [12-months, bi-weekly changes]; *indicates missing equipment/tube on ground (not analysed); EU/UK limit value of 40 µg/m3 (red dotted line) is also shown


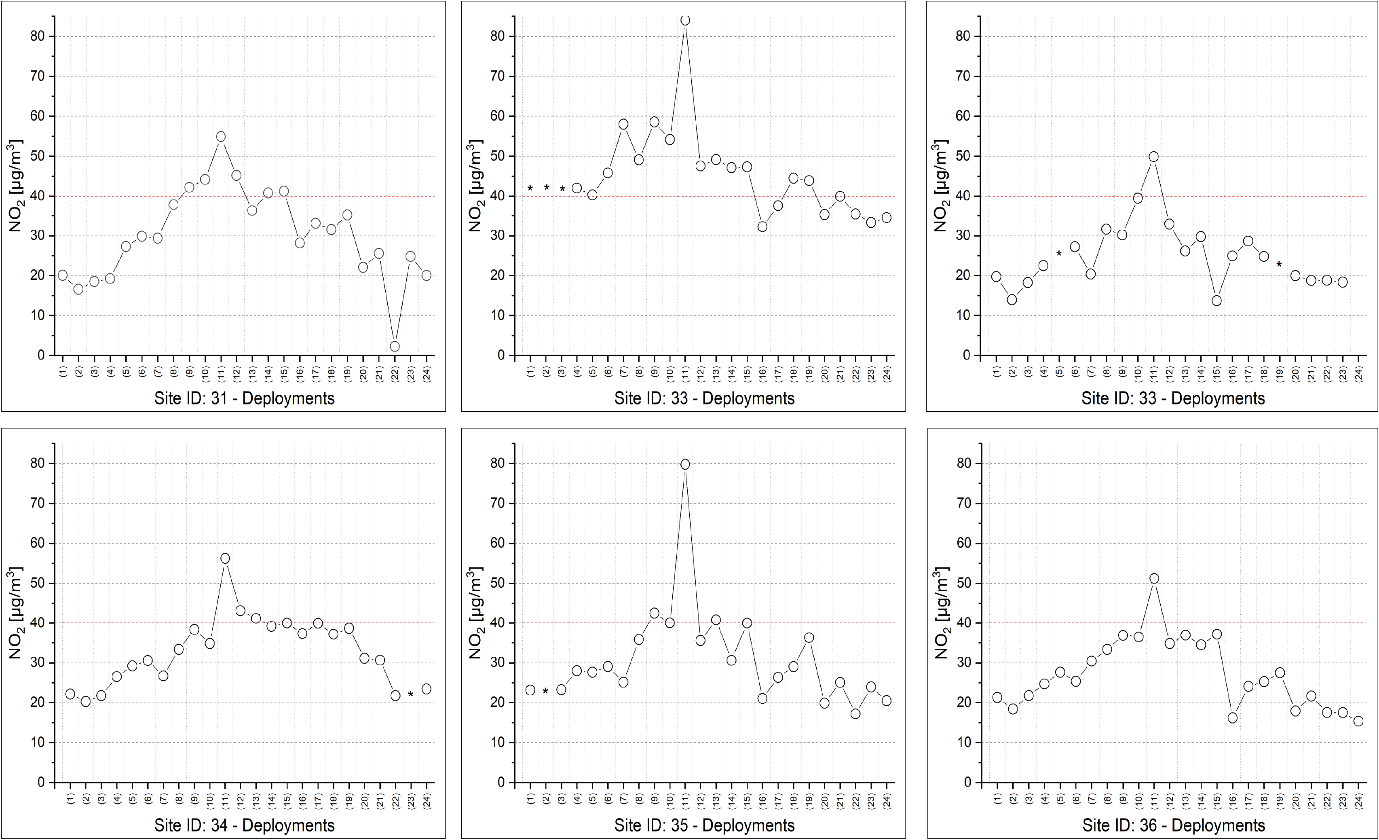


**Figure S10:** NO_2_ concentrations [µg m^-3^] recorded at sampling sites ID: 31 to ID: 36 for the deployment periods (1) to (24) [12-months, bi-weekly changes]; *indicates missing equipment/tube on ground (not analysed); EU/UK limit value of 40 µg/m^3^ (red dotted line) is also shown


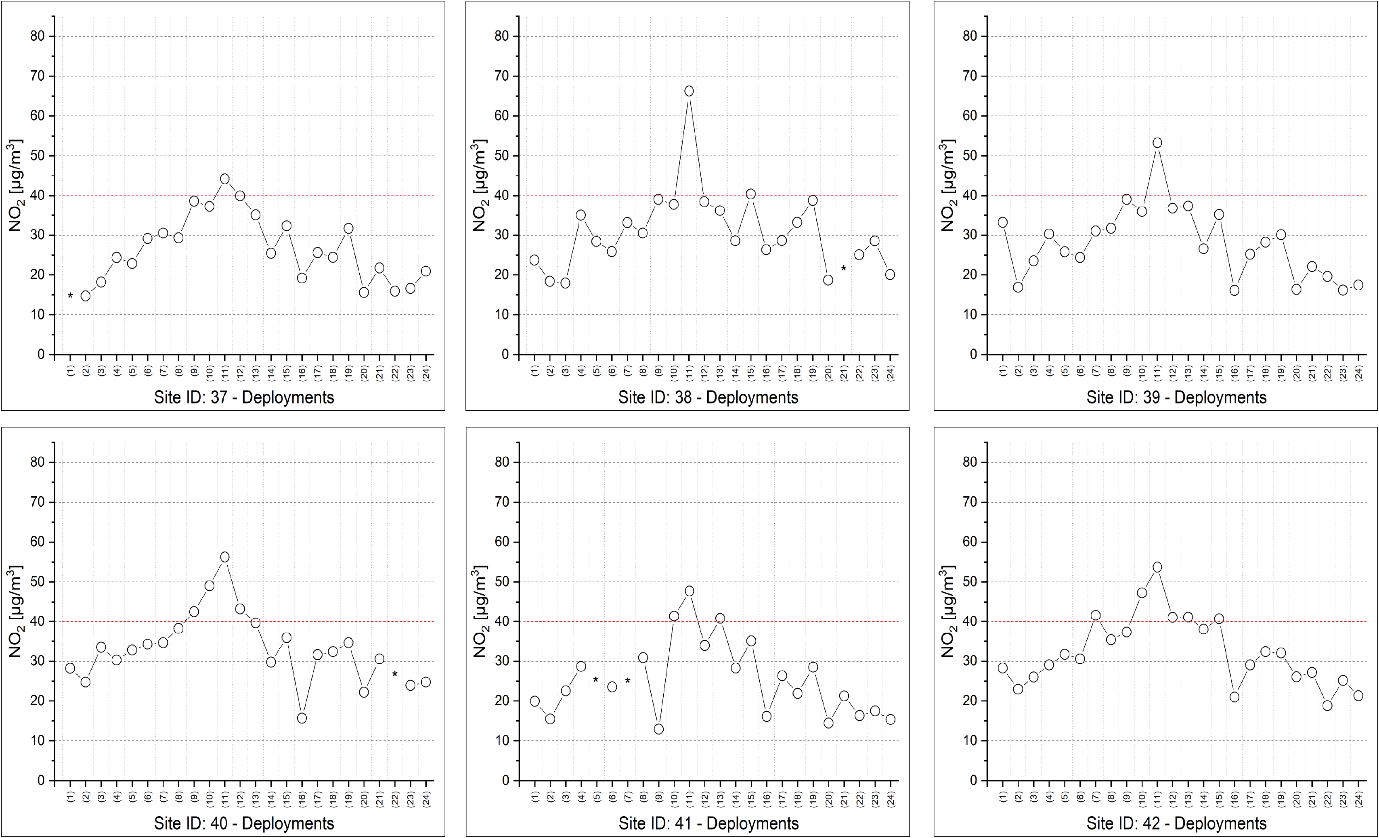


**Figure S11:** NO_2_ concentrations [µgm^-3^] recorded at sampling sites ID: 37 to ID: 42 for the deployment periods (1) to (24) [12-months, bi-weekly changes]; *indicates missing equipment/tube on ground (not analysed); EU/UK limit value of 40 µg/m^3^ (red dotted line) is also shown


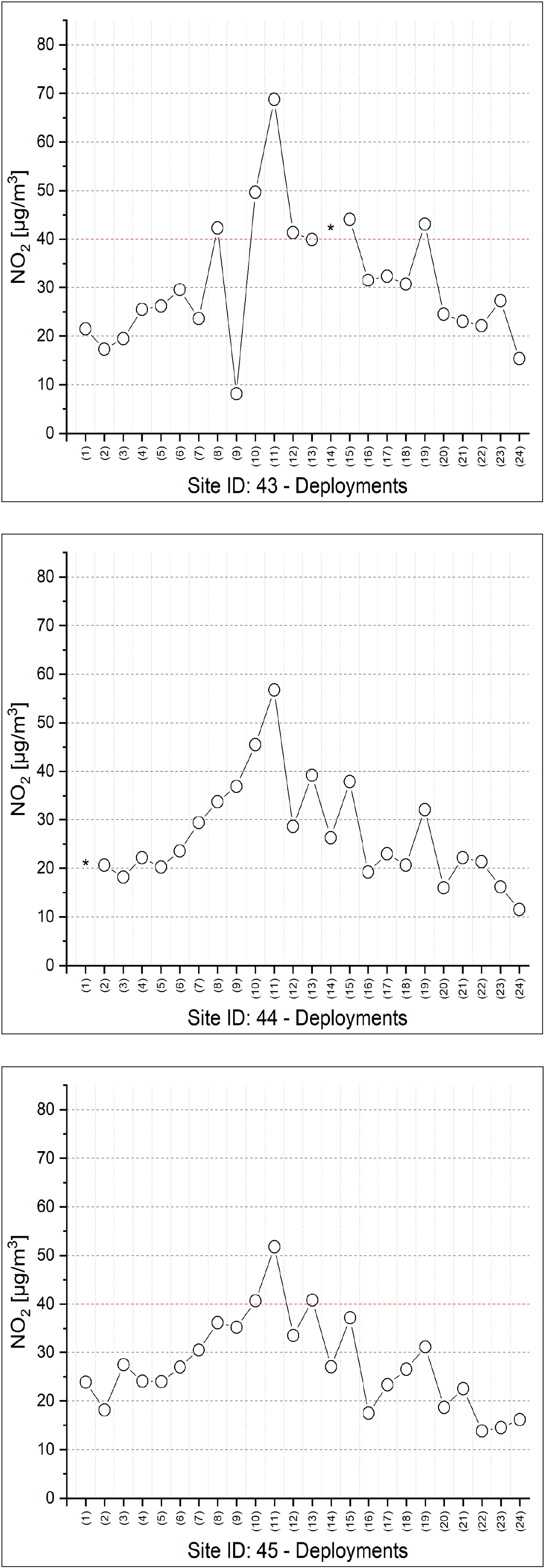


**Figure S12:** NO_2_ concentrations [µg m^-3^] recorded at sampling sites ID: 43 to ID: 45 for the deployment periods (1) to (24) [12-months, bi-weekly changes]; *indicates missing equipment/tube on ground (not analysed); EU/UK limit value of 40 µg/m^3^ (red dotted line) is also shown

**Table S6:** NO_2_ concentration ranges (minimum and maximum), 12-month average (± 1σ) and seasonal average (calculated from 6 consecutive 2-weekly measurements – 3 month intervals; ± 1 σ) for 45 diffusion tube deployment sites across Manchester city centre

| Site ID | Minimum NO_2_ [µg m^-3^] | Maximum NO_2_ [µg m^-3^] | 12-month NO_2_ [µg m^-3^] ± 1σ | Exceedances of EU/UK limit value in % (and total - N) | Seasonal NO_2_ [µg m^-3^] ± 1σ | | | |
| --- | --- | --- | --- | --- | --- | --- | --- | --- |
|  |  |  |  |  | **Summer** | **Autumn** | **Winter** | **Spring** |
| 1 | 28.45 | 68.54 | 43.95 ± 10.7 | 50% (N=12) | 37.97 ± 2.38 | 50.75 ± 10.3 | 49.14 ± 13.6 | 37.94 ± 7.70 |
| 2 | 18.79 | 52.50 | 33.09 ± 9.95 | 21% (N=5) | 31.25 ± 4.60 | 38.84 ± 6.09 | 36.51 ± 12.2 | 25.74 ± 6.15 |
| 3 | 21.99 | 60.07 | 39.54 ± 10.2 | 46% (N=11) | 36.21 ± 3.27 | 49.20 ± 5.32 | 39.99 ± 13.9 | 32.75 ± 8.10 |
| 4 | 17.08 | 48.94 | 28.91 ± 8.24 | 13% (N=3) | 25.46 ± 4.20 | 36.56 ± 9.38 | 32.02 ± 5.11 | 21.58 ± 4.49 |
| 5 | 20.09 | 59.09 | 37.37 ± 10.7 | 38% (N=9) | 33.16 ± 4.40 | 47.98 ± 8.96 | 38.65 ± 11.3 | 29.70 ± 8.59 |
| 6 | 31.05 | 62.63 | 43.81 ± 9.03 | 58% (N=14) | 40.23 ± 5.39 | 52.07 ± 7.00 | 46.47 ± 10.2 | 36.65 ± 5.87 |
| 7 | 23.91 | 54.92 | 35.67 ± 8.32 | 38% (N=9) | 31.64 ± 5.46 | 45.30 ± 5.37 | 35.66 ± 7.26 | 30.10 ± 6.21 |
| 8 | 14.69 | 43.60 | 27.94 ± 8.45 | 17% (N=4) | 21.98 ± 4.57 | 38.67 ± 5.54 | 29.50 ± 5.26 | 21.59 ± 4.15 |
| 9 | 21.01 | 65.27 | 41.43 ± 10.3 | 42% (N=10) | 33.95 ± 7.50 | 53.31 ± 10.2 | 39.41 ± 8.59 | 39.04 ± 2.42 |
| 10 | 16.55 | 41.59 | 29.35 ± 7.76 | 21% (N=5) | 24.01 ± 4.00 | 37.26 ± 5.08 | 32.72 ± 7.50 | 23.42 ± 3.70 |
| 11 | 18.75 | 45.24 | 29.58 ± 8.60 | 17% (N=4) | 24.91 ± 4.45 | 38.23 ± 5.74 | 31.99 ± 9.35 | 23.20 ± 5.30 |
| 12 | 11.96 | 48.24 | 28.16 ± 10.7 | 17% (N=4) | 22.22 ± 4.17 | 38.59 ± 6.58 | 30.71 ± 11.9 | 20.14 ± 7.11 |
| 13 | 24.05 | 62.30 | 40.18 ± 9.38 | 46% (N=11) | 36.01 ± 5.31 | 50.16 ± 7.53 | 40.86 ± 9.38 | 33.67 ± 6.35 |
| 14 | 21.37 | 47.67 | 32.08 ± 6.42 | 17% (N=4) | 29.00 ± 6.02 | 39.22 ± 4.39 | 31.39 ± 5.58 | 28.69 ± 4.00 |
| 15 | 25.57 | 58.93 | 37.61 ± 9.33 | 38% (N=9) | 32.60 ± 6.41 | 47.78 ± 7.02 | 41.24 ± 6.08 | 28.81 ± 3.23 |
| 16 | 23.30 | 52.91 | 33.44 ± 8.10 | 17% (N=4) | 27.60 ± 5.13 | 40.19 ± 7.89 | 35.81 ± 6.57 | 28.34 ± 5.89 |
| 17 | 23.96 | 61.59 | 41.55 ± 9.49 | 54% (N=13) | 33.21 ± 3.04 | 52.93 ± 6.08 | 40.20 ± 8.50 | 38.46 ± 6.44 |
| 18 | 29.36 | 53.75 | 39.51 ± 7.67 | 46% (N=11) | 35.72 ± 4.43 | 47.50 ± 4.24 | 39.76 ± 7.43 | 35.04 ± 7.72 |
| 19 | 26.85 | 61.63 | 41.79 ± 8.21 | 58% (N=14) | 37.74 ± 5.61 | 49.13 ± 6.80 | 43.46 ± 5.84 | 36.84 ± 8.99 |
| 20 | 14.48 | 55.58 | 30.40 ± 9.09 | 13% (N=3) | 26.86 ± 4.87 | 36.42 ± 13.6 | 33.17 ± 6.53 | 25.17 ± 5.40 |
| 21 | 19.18 | 46.01 | 28.04 ± 6.72 | 4% (N=1) | 23.75 ± 3.83 | 35.38 ± 7.40 | 30.56 ± 2.28 | 23.72 ± 5.42 |
| 22 | 7.35 | 54.74 | 33.76 ± 10.0 | 25% (N=6) | 32.01 ± 6.06 | 38.74 ± 16.5 | 36.44 ± 2.13 | 27.85 ± 8.08 |
| 23 | 22.59 | 47.02 | 32.64 ± 6.81 | 8% (N=2) | 29.73 ± 6.29 | 37.73 ± 7.30 | 34.57 ± 4.49 | 28.03 ± 5.45 |
| 24 | 25.92 | 56.26 | 36.30 ± 7.77 | 29% (N=7) | 32.32 ± 5.41 | 44.25 ± 7.11 | 36.79 ± 8.19 | 31.82 ± 3.44 |
| 25 | 24.22 | 64.15 | 41.70 ± 9.76 | 58% (N=14) | 37.45 ± 4.50 | 49.27 ± 7.98 | 45.03 ± 10.6 | 35.04 ± 9.21 |
| 26 | 10.65 | 39.73 | 23.28 ± 8.96 | 0% (N=0) | 18.45 ± 3.77 | 32.44 ± 6.86 | 25.87 ± 8.27 | 16.35 ± 6.69 |
| 27 | 22.50 | 63.46 | 35.24 ± 10.2 | 33% (N=8) | 28.90 ± 4.36 | 46.66 ± 9.85 | 37.42 ± 7.19 | 27.99 ± 6.17 |
| 28 | 16.92 | 48.39 | 29.68 ± 8.30 | 8% (N=2) | 22.28 ± 3.85 | 38.65 ± 6.27 | 32.77 ± 3.88 | 25.01 ± 6.91 |
| 29 | 13.43 | 44.87 | 26.66 ± 8.00 | 4% (N=1) | 22.79 ± 5.72 | 34.36 ± 6.71 | 29.28 ± 5.23 | 20.20 ± 6.49 |
| 30 | 30.87 | 68.98 | 50.00 ± 9.90 | 92% (N=22) | 47.32 ± 9.30 | 57.99 ± 6.99 | 48.72 ± 10.1 | 45.96 ± 10.4 |
| 31 | 2.26 | 54.92 | 30.25 ± 11.6 | 25% (N=6) | 21.91 ± 5.43 | 42.28 ± 8.47 | 35.19 ± 5.23 | 21.64 ± 10.8 |
| 32 | 32.23 | 84.05 | 45.70 ± 11.7 | 58% (N=14) | 42.71 ± 2.81 | 58.57 ± 13.3 | 42.97 ± 6.65 | 37.06 ± 4.04 |
| 33 | 13.71 | 49.89 | 25.23 ± 8.66 | 4% (N=1) | 20.32 ± 4.94 | 34.07 ± 9.91 | 24.65 ± 5.73 | 18.99 ± 0.69 |
| 34 | 20.27 | 56.26 | 33.21 ± 8.75 | 17% (N=4) | 25.08 ± 4.29 | 38.78 ± 10.2 | 39.16 ± 1.61 | 29.13 ± 6.80 |
| 35 | 17.16 | 79.79 | 31.33 ± 12.9 | 21% (N=5) | 26.22 ± 2.81 | 43.14 ± 18.9 | 31.32 ± 7.78 | 23.80 ± 6.77 |
| 36 | 15.34 | 51.25 | 27.25 ± 8.95 | 4% (N=1) | 23.19 ± 3.32 | 37.22 ± 7.27 | 29.03 ± 8.52 | 19.58 ± 4.37 |
| 37 | 14.69 | 44.24 | 26.68 ± 8.50 | 4% (N=1) | 21.86 ± 5.60 | 36.64 ± 5.74 | 27.02 ± 5.75 | 20.41 ± 6.10 |
| 38 | 17.96 | 66.27 | 31.26 ± 10.4 | 8% (N=2) | 24.89 ± 6.44 | 40.87 ± 12.9 | 32.23 ± 5.39 | 26.24 ± 8.06 |
| 39 | 16.13 | 53.25 | 28.01 ± 9.09 | 4% (N=1) | 25.67 ± 5.67 | 37.98 ± 8.09 | 28.10 ± 7.60 | 20.31 ± 5.28 |
| 40 | 15.69 | 56.26 | 33.42 ± 8.95 | 17% (N=4) | 30.62 ± 3.67 | 43.99 ± 7.73 | 30.84 ± 8.22 | 27.20 ± 5.22 |
| 41 | 12.88 | 47.74 | 25.40 ± 9.75 | 13% (N=3) | 22.02 ± 4.86 | 33.36 ± 13.2 | 28.09 ± 8.89 | 18.90 ± 5.27 |
| 42 | 18.82 | 53.75 | 32.41 ± 8.79 | 25% (N=6) | 28.09 ± 3.20 | 42.73 ± 6.77 | 33.71 ± 7.85 | 25.10 ± 4.63 |
| 43 | 8.16 | 68.78 | 30.75 ± 13.3 | 25% (N=6) | 23.24 ± 4.60 | 38.98 ± 21.0 | 35.73 ± 5.95 | 25.89 ± 9.32 |
| 44 | 11.51 | 56.76 | 27.01 ± 10.7 | 8% (N=2) | 20.96 ± 2.01 | 38.48 ± 10.85 | 27.70 ± 8.75 | 19.89 ± 7.17 |
| 45 | 13.84 | 51.75 | 27.56 ± 9.46 | 13% (N=3) | 24.07 ± 3.31 | 37.98 ± 7.52 | 28.72 ± 8.75 | 19.48 ± 6.52 |

**Additional References, referred to within supplementary information:**

Air Quality England. (2018a). *Air Pollution Report - Manchester Piccadilly (MAN7)*. Manchester.

Air Quality England. (2018b). *Air Pollution Report - Manchester Oxford Road (MAN1)*. Manchester.

Bermejo-Orduna, R., McBride, J. R., Shiraishi, K., Elustondo, D., Lasheras, E., & Santamaría, J. M. (2014). Biomonitoring of traffic-related nitrogen pollution using *Letharia vulpina* (L.) Hue in the Sierra Nevada, California. *Science of the Total Environment*, *490*, 205–212. https://doi.org/10.1016/j.scitotenv.2014.04.119

Boltersdorf, S. H., Pesch, R., & Werner, W. (2014). Comparative use of lichens, mosses and tree bark to evaluate nitrogen deposition in Germany. *Environmental Pollution*, *189*, 43–53. https://doi.org/10.1016/j.envpol.2014.02.017

Britter, R. E., & Hanna, S. R. (2003). Flow and Dispersion in Urban Areas. *Annual Review of Fluid Mechanics*, *35*(1), 469–496. https://doi.org/10.1146/annurev.fluid.35.101101.161147

Buccolieri, R., Sandberg, M., & Di Sabatino, S. (2010). City breathability and its link to pollutant concentration distribution within urban-like geometries. *Atmospheric Environment*. https://doi.org/10.1016/j.atmosenv.2010.02.022

Defra. (2008). *Diffusion Tubes for Ambient NO2 Monitoring: Practical Guidance for Laboratories and Users*. *ED48673043*. Didcot.

DfT. (2017). Road traffic statistics. https://www.gov.uk/government/publications/road-traffic-estimates-great-britain-jan-to-mar-q1-2014%5Cnhttps://www.gov.uk/government/collections/road-traffic-statistics

Digimap - Ordnance Survey. (2016). OS Open Roads. https://www.ordnancesurvey.co.uk/business-and-government/products/os-open-roads.html. Accessed 17 June 2017

Digimap - Ordnance Survey. (2017). OS Building Heights (Alpha). https://digimap.edina.ac.uk/webhelp/os/data_information/os_products/os_building_heights.htm. Accessed 11 December 2018

Gilbert, N. L., Woodhouse, S., Stieb, D. M., & Brook, J. R. (2003). Ambient nitrogen dioxide and distance from a major highway. *Science of the Total Environment*, *312*(1–3), 43–46. https://doi.org/10.1016/S0048-9697(03)00228-6

Hertel, O., & Goodsite, M. E. (2009). Urban Air Pollution Climates throughout the World. *Air Quality in Urban Environments*, 1–22. https://doi.org/10.1039/9781847559654-00001

Laffray, X., Rose, C., & Garrec, J. P. (2010). Biomonitoring of traffic-related nitrogen oxides in the Maurienne valley (Savoie, France), using purple moor grass growth parameters and leaf 15N/14N ratio. *Environmental Pollution*, *158*(5), 1652–1660. https://doi.org/10.1016/j.envpol.2009.12.005

Lo, K. W., & Ngan, K. (2015). Characterising the pollutant ventilation characteristics of street canyons using the tracer age and age spectrum. *Atmospheric Environment*, *122*, 611–621. https://doi.org/10.1016/j.atmosenv.2015.10.023

Massman, W. J. (1998). A Review of the Molecular Diffusivities of H2O, CO2, CH4, CO, O3, SO2, NH3, N2O, NO, and NO2 in Air, O2 and N2 near STP. *Atmospheric Environment*, *32*(6), 1111–1127.

Niepsch, D. (2019). *Application of lichen-biomonitoring to assess spatial variability of urban air quality in Manchester (UK)*. Manchester Metropolitan University.

Shen, J., Gao, Z., Ding, W., & Yu, Y. (2017). An investigation on the effect of street morphology to ambient air quality using six real-world cases. *Atmospheric Environment*, *164*, 85–101. https://doi.org/10.1016/j.atmosenv.2017.05.047

Whitworth Meterological Observatory - Data Archive. (2018). http://whitworth.cas.manchester.ac.uk/2018/. Accessed 16 October 2018
